# STRING-ing together protein complexes: corpus and methods for extracting physical protein interactions from the biomedical literature

**DOI:** 10.1093/bioinformatics/btae552

**Published:** 2024-09-14

**Authors:** Farrokh Mehryary, Katerina Nastou, Tomoko Ohta, Lars Juhl Jensen, Sampo Pyysalo

**Affiliations:** TurkuNLP Group, Department of Computing, University of Turku, Turku 20014, Finland; Novo Nordisk Foundation Center for Protein Research, University of Copenhagen, Copenhagen 2200, Denmark; Textimi, 1-37-13 Kitazawa, Tokyo, Setagaya-ku 155-0031, Japan; Novo Nordisk Foundation Center for Protein Research, University of Copenhagen, Copenhagen 2200, Denmark; TurkuNLP Group, Department of Computing, University of Turku, Turku 20014, Finland

## Abstract

**Motivation:**

Understanding biological processes relies heavily on curated knowledge of physical interactions between proteins. Yet, a notable gap remains between the information stored in databases of curated knowledge and the plethora of interactions documented in the scientific literature.

**Results:**

To bridge this gap, we introduce ComplexTome, a manually annotated corpus designed to facilitate the development of text-mining methods for the extraction of complex formation relationships among biomedical entities targeting the downstream semantics of the physical interaction subnetwork of the STRING database. This corpus comprises 1287 documents with ∼3500 relationships. We train a novel relation extraction model on this corpus and find that it can highly reliably identify physical protein interactions (F1-score = 82.8%). We additionally enhance the model’s capabilities through unsupervised trigger word detection and apply it to extract relations and trigger words for these relations from all open publications in the domain literature. This information has been fully integrated into the latest version of the STRING database.

**Availability and implementation:**

We provide the corpus, code, and all results produced by the large-scale runs of our systems biomedical on literature via Zenodo https://doi.org/10.5281/zenodo.8139716, Github https://github.com/farmeh/ComplexTome_extraction, and the latest version of STRING database https://string-db.org/.

## 1 Introduction

The study of physical protein interactions forms a basis for understanding biological processes. These interactions are captured from experimental data (Licata *et al.* 2012, Orchard *et al.* 2014, Oughtred *et al.* 2021) or scientific articles (Meldal *et al.* 2019, Giurgiu *et al.* 2019, Gillespie *et al.* 2022) and are provided as periodically curated databases.

These curation efforts have been complemented by co-occurrence-based text mining in protein interaction databases, such as STRING (Szklarczyk *et al.* 2023) and HumanNet (Kim *et al.* 2021), to obtain more comprehensive networks. While this approach is powerful for linking molecules that function together, the fact that proteins are mentioned together in text is not enough to infer that they also physically interact. Thus, earlier versions of STRING (Franceschini *et al.* 2012) employed a rule-based system to help with the extraction of such interactions.

In the field of biomedical natural language processing (BioNLP), the past two decades have witnessed significant progress, driven by the development of more sophisticated and accurate deep learning-based methodologies (Milošević and Thielemann 2023) and manually annotated corpora. Building deep learning-based NLP and text mining systems typically involves a two-step process: self-supervised pretraining of a model on an unannotated corpus with a language modeling objective, and fine-tuning the pretrained model on manually annotated data for a specific downstream task (e.g. relation extraction). Models based on the transformer architecture (Vaswani *et al.* 2017) such as BERT (Devlin *et al.* 2019) have been particularly effective, combining efficient training of large-scale models using GPU acceleration with state-of-the-art performance across a broad range of tasks. Recently, large language models (LLMs) trained for text generation [e.g. GPT (OpenAI *et al.* 2024) or LLaMA (Touvron *et al.* 2023) models] have been explored for NLP tasks beyond question answering, such as named entity recognition (NER; Wang *et al.* 2023) and relation extraction (RE; Wan *et al.* 2023). In this approach, the focus is on developing zero/few-shot prompting techniques to build text mining and NLP systems, requiring at most a few training examples. However, when it comes to the biomedical domain, recent extensive benchmarks show that this approach performs on average 30% worse than BERT-based models (Jimenez Gutierrez *et al.* 2022, Chen *et al.* 2023, Jahan *et al.* 2023).

The effectiveness of the original BERT and other biomedical domain-specific BERT models (Lee *et al.* 2019, Lewis *et al.* 2020) depends on the availability of manually annotated corpora for fine-tuning. Generating these corpora requires expert knowledge, making it a costly endeavor. Even when these corpora are available, their seamless integration in downstream tasks is far from straightforward. Existing manually annotated corpora of physical protein interactions either cannot be grounded in text (Krallinger *et al.* 2008), the manually annotated interactions are limited to sentence-level annotations, or they focus solely on human (Bunescu *et al.* 2005, Nédellec 2005, Fundel *et al.* 2006; Pyysalo *et al.* 2007, Kim *et al.* 2009, Pyysalo *et al.* 2011). Moreover, the different definitions of complex formation among these corpora (Pyysalo *et al.* 2008) pose a substantial challenge in interoperability. For example, some corpora (Pyysalo *et al.* 2007, Kim *et al.* 2008, 2009, Pyysalo *et al.* 2011) include binding of proteins to DNA elements in their definition whereas some others do not (Fundel *et al.* 2006, Krallinger *et al.* 2008, Bunescu *et al.* 2005). In addition, these corpora differ in their definitions and annotations of named entities (Pyysalo *et al.* 2008). Considering all the limitations above, leveraging existing data for transfer learning for RE of protein–protein interactions quickly becomes a nonviable option given either the varied definitions for both relations and named entities, the limited scope to one organism, the lack of grounding in text, or the fact that most available corpora contain only sentence-level annotations.

In this study, our primary objective was to develop a system to support the relation extraction of physical protein–protein interactions from the literature for the STRING database. For this purpose, we present ComplexTome, a new corpus annotated with complex formation relationships between biomedical entities. In this corpus, we overcome previous limitations, by targeting specific downstream semantics, and providing document-level annotations, grounded in text and not limited to a single organism. The annotated relations in ComplexTome include cases where the associated constituents are proteins, protein complexes, protein families, or chemicals, but not DNA elements. We have also built a relation extraction pipeline, trained it on ComplexTome to extract such relationships from the openly accessible biomedical literature, and created a trigger word detection system to aid in interpreting the results. We provide the corpus, code, and all results produced by the large-scale runs of our system via Zenodo https://doi.org/10.5281/zenodo.8139716, Github https://github.com/farmeh/ComplexTome_extraction, and the latest version of STRING https://string-db.org/.

## 2 Materials and methods

### 2.1 The ComplexTome corpus

#### 2.1.1 Document selection for corpus annotation

The selection of documents for ComplexTome involved a three-step approach of selecting documents from

1. *Existing corpora*: Initially, we explored established corpora, particularly the BioNLP ST 2009 training and development datasets (Kim *et al.* 2009). From these datasets, we identified and selected a total of 135 abstracts featuring instances of complex formation events, from which we could potentially extract documents with the desired relationship type present. As the definition and annotation of binding events for the BioNLP ST 2009 corpus were not compatible with the relationship annotation we were aiming for in ComplexTome, all existing annotations were discarded and the documents selected were reannotated from scratch. A comparison of binarized binding events from this corpus with our final annotations in ComplexTome showed that these two sets had less than 50% overlap, which supports our decision to reannotate everything from scratch for our corpus. Details on this comparison are provided in Supplementary Material Section S1.
2. *Resources enriched in positive relationships*: To broaden the corpus, and considering the limitations of other corpora discussed in Introduction section, we decided to expand to other sources of documents for annotation, where relations of interest were expected to be present. Specifically, we curated 400 abstracts extracted from a collection of 66 757 publications used as evidence to support physical or genetic interaction entries in the BioGRID (Oughtred *et al.* 2021), IntAct (Orchard *et al.* 2014), and MINT (Licata *et al.* 2012) interaction databases. Additionally, 400 paragraphs extracted from 12 577 articles available as PubMed Central Open Access (PMC-OA) full-text articles were selected from the same databases. Documents used to annotate more than 20 interactions in the databases were removed from the selection pool as these interactions are usually extracted from supplementary tables and not the actual text of scientific articles.
3. *Resources enriched in negative relationships*: We also selected 300 abstracts extracted from 21 941 papers used for pathway annotation in the Reactome pathway knowledgebase (Gillespie *et al.* 2022) and 50 abstracts extracted from 15 319 papers in BioGRID filtered to include only experimental associations for genetic interactions.

During steps 2 and 3, we used a dictionary-based named entity recognition (NER) method (Jensen 2016), to detect protein entities within the large document pools and restricted the selection to abstracts containing a moderate number of detected protein entities (between 2 and 40). We selected documents with at least two entities as this is a prerequisite for relation extraction, while we put a threshold to 40 entities as our initial observations, showed that documents with more entities than that are usually documents with long lists of named entities, with no relations of interest present therein. To prevent over-representation of commonly mentioned protein entities and entities from specific species, abstracts featuring highly mentioned proteins were limited to comprise at most 2% of the selected documents. All documents in ComplexTome were annotated using the BRAT rapid annotation tool (Stenetorp *et al.* 2012).

#### 2.1.2 Named entity annotation

We annotated four named entity (NE) types, namely Gene or Gene Product (Protein hereafter), Chemical which encompasses standalone chemicals that are not part of larger chemical/protein entities, Complex for entities describing stable assemblies of two or more macromolecules, in which at least one component is a protein, and Protein_Family, for entities which represent an evolutionarily conserved group of gene/proteins or a group of entities with the same function. To assist the manual annotation process of NEs, we used automated NER (Jensen 2016) for the detection of Protein entities in our corpus.

In the scientific literature, it is common to encounter alternative names referring to identical entities. We have systematically recorded these equivalent names in ComplexTome. This practice is crucial for accurate evaluation, as it allows relationships stemming from either entity to be recognized as valid (Kim *et al.* 2009). To allow for easy filtering of the NEs, we used five entity attributes to mark NEs in our corpus: “Mutant”, “Fusion”, “Non-coding”, “Small protein post-translation modification”, and “Blocklisted” (for a description of these attributes, refer to our annotation documentation https://katnastou.github.io/annodoc-physical-protein-interaction-corpus/).

#### 2.1.3 Relationship annotation

**Figure 1.**
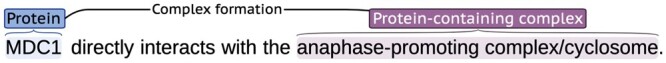
Illustration of the relationship representation in ComplexTome. A Complex_formation relationship between a Protein (“MDC1”) and a Complex (“anaphase-promoting complex/cyclosome”) participant has been identified by the annotators in the example above.

In ComplexTome, we identified explicit mentions of physical protein interactions and annotated these as undirected binary relationships with the type Complex_formation. We added annotations for any statement implying the existence of a complex, but not statements explicitly denying the formation of a complex. A relationship annotation example is shown in Fig. 1.

The annotations were performed by two domain experts, ensuring accurate relationship identification. Moreover, to establish consistent annotation guidelines and maintain annotation quality, we conducted an interannotator agreement (IAA) analysis by independently annotating a set of abstracts during the two initial steps of document selection. The process encompassed four rounds of independent annotations with at least 20 documents selected and annotated per round. We calculated Cohen’s kappa (Cohen 1960) after each round of IAA to assess annotation consistency and corpus quality. For comprehensive details on the annotation rules followed to produce the corpus and on the annotation process itself, we refer readers to the annotation documentation provided to the annotators (see Section 2.1.2). This documentation served as a reference to ensure a shared understanding of the rules among annotators and contributed to maintaining the overall quality of annotations.

### 2.2 Relation extraction system

We have developed a relation extraction system that is capable of extracting Complex_formation relations between Protein named entities, as stated in biomedical texts. We cast the task of relation extraction as binary classification, predicting whether a Complex_formation relation has been stated for the two candidate NEs in the given input text (i.e. a positive label) or not (i.e. a negative label).

The system is based on deep neural networks with an architecture consisting of a pretrained transformer encoder, followed by a decision layer with a softmax activation function. The system can utilize existing pretrained language models that are currently available in the Hugging Face repository (https://huggingface.co/), allowing us to benchmark different models and use the best available model for the task. Training, validation, and prediction files can be provided to the system in BRAT standoff format (as well as a custom JSON format). The system supports two input representation methods: marking and masking the NEs (Mehryary *et al.* 2020) and it can be trained with a wide variety of hyper-parameters including maximum sequence length (MSL), learning rate, mini-batch size, number of training epochs, and random seed. During the training on the ComplexTome training data, pretrained weights of the encoder are fine-tuned, while randomly initialized weights (such as the weights of the decision layer) are learned from scratch. After each training epoch, evaluation metrics are calculated on the development set and used for hyper-parameter optimization. We do not use any early stopping rule, instead, we train a network for the specified number of epochs (given as a hyper-parameter) but use model weights of the epoch which has yielded the highest F1-score. For implementation, we use Python programming language with TensorFlow and transformers libraries. Supplementary Material Section S3.1 provides more information about the relation extraction system architecture and the transformer models used in this work and why they were chosen.

#### 2.2.1 Preprocessing, input representation, and example generation

As biomedical texts typically contain more than one candidate NE pair and can be very long, not fitting within the maximum input tokens of transformer models, we process each input document as follows.

1. *Marking or masking the entities*: To inform the classifier which two particular NEs constitute a pair at a time for label prediction, we transform the text by encoding the entities in the input document, either using a marking approach or a masking approach, utilizing the language model’s “unused” tokens for this aim. Additionally, we prepend a [CLS] token, representing the snippet start, and append a [SEP] token to the tokens, representing the end of snippet, before feeding them to neural network models.
2. *Tokenization, distance calculation, and example generation*: For each candidate NE pair, after transforming the text (marking/masking), we tokenize the text, and if the two entities can fit into a window with a size less than or equal to the specified MSL, we generate a machine learning example for the pair. The pair can have a positive, or a negative label (for training) or can be unlabeled (during prediction).

Our preprocessing approach provides two benefits: Since we are not performing any sentence boundary detection, the system is able to train with and predict cross-sentence relations. In addition, there will be no problem in dealing with long texts, since we rely on a window (sequence of sub-tokens) that can always be fed to the transformer encoder. Supplementary Material Section S2 provides more details and a few examples for all steps described here.

#### 2.2.2 Baseline system

For comparison, we also developed a baseline system with the same architecture as the main relation extraction system, except using the original BERT-base-cased transformer-encoder (Devlin *et al.* 2019). We use the same training and evaluation method and experimental setup for the baseline system and the main relation extraction system.

#### 2.2.3 Experimental setup

We performed a document-based split of ComplexTome to generate separate training, development, and test sets, which consisted of 772, 258, and 257 documents, respectively. We train the system on the training set and optimize it on the development set. We use a grid search to try different transformer models and find the optimal values of hyper-parameters. To minimize the effect of initial random weights on evaluation scores (Mehryary *et al.* 2016), we repeat each experiment four times and compare different experiments based on the average and standard deviation of the F1-scores. Each *experiment* consists of training a relation extraction system with different initial random weights but with the same transformer encoder weights and the exact set of hyper-parameters on the training set and evaluating the model on the development set. The held-out test set was only used once for the final evaluation of our best system. Supplementary Material Section S3.2 provides a list of tested models and hyper-parameters.

Even though our corpus contains four NE types (Protein, Chemical, Complex, and Protein_Family), and Complex_formation relationships can occur between any two NEs, for the real-world application for which the system was intended for, i.e. extracting Protein-Protein interactions for STRING database v12, the system had to deal with texts including only Protein entities, which are not “blocklisted”. Hence, to have a realistic optimization approach for large-scale prediction, we filtered out all non-Protein entities and Protein entities with the “blocklisted” attribute (and their relations) from the development and test sets. In contrast, in order to assess whether we could leverage the full potential of relationship annotation in our corpus, during training we performed several experiments with different training schemes. More details on the experimental setup are provided in Supplementary Material Section S4.

### 2.3 Trigger detection system

While detecting Complex_formation relations between biomedical entities in the scientific literature is a task of paramount importance to the scientific community, it is even more beneficial if, for each Complex_formation relation extracted from a text snippet, there was a system that could also highlight the most important word or phrase in the text snippet that explicitly or implicitly denotes the relation. In the BioNLP community, such words or phrases are called “trigger words” (hereafter “triggers”) and were popularized as part of the biomedical event representation of the BioNLP Shared Task 2009 on Event Extraction (Kim *et al.* 2009). In the context of Complex_formation extraction, triggers can be as simple as “bind” or as complicated as “tandem affinity purification”.

Traditionally, “trigger detection” (automatic recognition of the keywords/phrases behind the extraction of events or relations) has been defined as a supervised NER task, relying on manually annotated data for training and evaluation. However, with the help of model explanation methods, such as *Integrated Gradients* (Sundararajan *et al.* 2017) and *SHAP (SHapley Additive exPlanations) values* (Lundberg and Lee 2017), we aim to automatically find the triggers in an unsupervised fashion. The general idea here is to apply such methods to calculate and assign a score to each token of the input with regard to its contribution to the predicted label (Complex_formation in our case), and by ranking the tokens based on their scores, we can obtain the token(s) with the highest contribution to the label for the extracted relations. We hypothesize that these tokens will frequently correspond to the triggers. Naturally, this is done only for Protein-Protein pairs with a positive label, i.e. when the model has predicted that there is a Complex_formation relationship between two candidate NEs in a given input text, as by definition, triggers are the words or phrases that explicitly state or imply the existence of a relationship between two NEs.

While using model explanation methods to attribute importance scores to input tokens has previously been applied in areas like sentiment analysis (e.g. identifying keywords in movie reviews; Dewi *et al.* 2022), its application to automatic trigger detection, as presented in this manuscript, is novel.

#### 2.3.1 Experimental setup

Model explanation methods are generally used to provide useful insights about how a *trained* model works. Therefore, the design and development of the trigger detection system started once we had obtained the final neural network model for Complex_formation extraction.

While such methods are unsupervised, not requiring any manually annotated data for training, we still needed manually annotated data to evaluate and compare approaches, since there is no guarantee that the token(s) with the highest contribution to a positive label is the trigger we aim to recognize. Therefore, we focused on the *positive pairs* of the ComplexTome development set. We first split this set into two equally sized sets (based on the number of documents), hereafter a *trigger development set*, and a *trigger test set*. We then excluded those infrequent positive examples from the two sets that do not fit into a window of 128 tokens, since this is the best MSL found during hyper-parameter optimization, and our best model has been trained with this restriction for real-world application. After filtering, there are 284 and 275 positive Protein-Protein pairs in the trigger development and test set, respectively, for which we aim to recognize triggers.

The two sets were subsequently given for annotation to an expert, with the annotation task of highlighting trigger text spans for each positive pair. If two or more text spans were considered as equivalently valid triggers, they were all annotated as triggers for a Protein-Protein pair, but we emphasize that correctly recognizing and showing only one of the triggers to the end-user in such cases is sufficient. The section “Trigger word annotation” in the annotation guidelines (https://katnastou.github.io/annodoc-physical-protein-interaction-corpus/) provides details with interesting examples.

#### 2.3.2 Trigger detection methods

We experiment with two commonly used model explanation methods:

1. *Layer integrated gradients* **(LIG)**, as implemented in the Captum library (https://captum.ai/), which is a variant of the integrated gradients method that assigns an importance score to a desired layer’s outputs of a trained neural network model, for every token in the input snippet.
2. ***SHapley Additive exPlanations* (SHAP) *values*** (https://github.com/shap/shap), which is a method commonly used for explaining the predictions of a machine learning model. SHAP calculates a value that represents the contribution of each token in the input snippet to the model outcome, thus the values reflect the importance of each token with regard to the label.

Our best relation extraction model was obtained by fine-tuning a pretrained RoBERTa model (Lewis *et al.* 2020) on the ComplexTome training set. This is consistent with the top-performing teams in the recent DrugProt relation-extraction track of BioCreative VII using pre-trained RoBERTa models (Miranda-Escalada *et al.* 2023). By feeding trigger development set examples as input to the model and applying the LIG method on the outputs of the embedding layer and the 24 hidden RoBERTa layers in this model, we obtain 25 vectors for each development set example. By discarding [CLS], [SEP], and “unused” tokens and then simply choosing the token with the highest score in each vector as the predicted trigger, we obtain 25 different predictions for each development set example. Therefore, we form 25 different prediction sets of the development set (each based on a particular layer in the model), which can further be evaluated against the gold standard. Similarly, by applying the SHAP method, we obtain one set of predictions. Initial experiments showed slight differences when feeding the inputs *with* and *without* the [CLS] and [SEP] tokens. Therefore, we tried both types of inputs for the SHAP method. In total, we obtained 27 predicted sets for the development set triggers, which we then compared against the gold standard and calculated evaluation scores. To further improve the results, we also defined a set of post-processing heuristic rules, targeting and removing irrelevant tokens from the vectors before choosing the tokens with the highest score as predicted triggers. Supplementary Material Section S5 provides further details about our trigger detection methods.

#### 2.3.3 Baseline method

For comparison, we also develop a simple baseline method. In this method, we first obtain the list of all words or phrases that are highlighted as triggers in the trigger development set. Then, for each Complex_formation relation in the trigger development set, we define two windows around the two candidate NEs (the window size in sub-tokens is an optimizable parameter, ranging from 1 to the maximum possible value for the trigger development set). Based on the selected window size, for each word or phrase in the list, we search the windows with regular expressions, and if we can match a word or phrase against a span in the texts of two windows, we annotate the span as a recognized trigger.

## 3 Results and discussion

### 3.1 Corpus statistics

ComplexTome is a high-quality corpus, supported by the fact that we attained over 90% agreement over four rounds of IAA, with a Cohen’s kappa of 0.91 in the last round of IAA—an almost perfect agreement between the two annotators (McHugh 2012). ComplexTome comprises 1287 documents with ∼300 000 words. There are 3486 Complex_formation relationships in the corpus, ensuring a rich and diverse collection of relations for training neural network models for the relation extraction task. Over 96% of these relationships are intra-sentence (i.e. within sentence boundaries), while less than 4% cross sentence boundaries. This is in line with statistics from other publicly available biomedical corpora with document-level annotations, where cross-sentence relations constitute <5% of the relations annotated therein (Björne *et al.* 2009, Mehryary *et al.*, 2018, Miranda-Escalada *et al.* 2023). There is a large number of NEs in the corpus, namely 20 228 Protein, 2185 Chemical, 1500 Complex, and 3019 Protein_Family entities. Notably, we gave the “Blocklisted” attribute to 795 entities during annotation and later properly filtered them out for the training and development of the relation extraction model. Supplementary Material Section S6 presents the distribution of NEs and relations in the ComplexTome corpus.

### 3.2 Relation extraction

#### 3.2.1 System evaluation

We used grid search to find the optimal values of hyper-parameters and compare different pre-trained transformer encoders, using the methodology described above. Our best result was achieved using the RoBERTa-large-PM-M3-Voc encoder (Lewis *et al.* 2020), a 24 layer RoBERTa-based language model, pretrained on biomedical and clinical texts, and using the following hyper-parameter values: MSL = 128, lr = 3e−6, training_epochs = 11, minibatch_size = 5. This experiment resulted in 86.8% average precision, 82.1% average recall, and 84.3% average F1-score on the ComplexTome development set. Table 1 shows the details of the four models used in this experiment and the evaluation scores measured on the development set (Table 1).

**Table 1.** Performance of the best experiment on the ComplexTome development set.^a^

	Precision (%)	Recall (%)	F1-score (%)
Model-1	88.8	79.4	83.8
Model-2	82.8	83.6	83.2
Model-3	86.9	82.4	84.6
Model-4	88.5	82.8	**85.5**
Average	86.8	82.1	84.3
std	2.8	1.8	1.0

a The best model (highlighted in gray, with F1-score = 85.5%) is used for large-scale prediction on the entire literature.

**Table 2.** Comparison of the baseline system with the main relation extraction system on the development set.^a^

	Precision (%)	Recall (%)	F1-score (%)
Baseline avg (std)	76.4 (3.8)	74.3 (2.5)	75.3 (1.9)
Main avg (std)	86.8 (2.8)	82.1 (1.8)	84.3 (1.0)

a The table shows the average and standard deviation of the scores (for the four best models) in each approach.

Our best model achieves 85.5% F1-score on the development set and 87.3% precision, 78.8% recall and 82.8% F1-score on the held-out test set. We have selected this particular model for large-scale execution and for providing the text-mined associations for the physical subnetwork of STRING v12.

#### 3.2.2 Baseline evaluation and comparison

Similarly to the main relation extraction system, we used grid search and found MSL = 128, lr = 5e-6, training_epochs = 12, and mini-batch size = 5 to be the optimal hyper-parameter values, resulting in the highest average F1-score on ComplexTome development set when using BERT-base-cased transformer model. Table 2 shows the comparison of the baseline system with the main relation extraction system on the development set.

As shown in Table 2, while the baseline system with 75.3% average F1-score has a moderately good performance on the task, it is 9 percentage points behind the main relation extraction system (84.3% average F1-score). Since both systems use the same training and evaluation setup, this shows how selecting a good pretrained transformer encoder can improve the performance on the task. For a detailed comparison between RoBERTa and BERT models, and to obtain more information about the transformer models used in this work, refer to Supplementary Material Section S3.

#### 3.2.3 Manual error analysis

We present an analysis of the errors produced by the best relation extraction model on the test set, grouped into categories in Table 3. For a detailed overview of all errors, refer to Supplementary Material Section S7. We provide instructions on comparing the documents in the annotated corpus and the model predictions in a BRAT server in our annotation guidelines. We also provide views of these comparisons as image files via Zenodo.

**Table 3.** Error analysis for the relation extraction system.

Error type	Count		
	FP	FN	Total
Ambiguous keyword	18	26	44
Rare keyword	2	23	25
Convoluted text excerpt	15	39	54
Co-reference resolution	11	9	20
Annotation error	11	8	19
Total	57	105	162

FN, False negative; FP, False positive.

We identified five error categories, with none appearing to be the primary cause of errors observed in the test set. The first category is “ambiguous keyword”, which involves words like “association” that can describe both physical interactions and other types of relationships. This makes it hard to assign an accurate relationship label, resulting in both FPs and FNs. “Rare keyword” pertains to relationships between NEs that annotators have identified based on their biological knowledge, but which are indicated by phrases or words that are seldom encountered in the literature (e.g. non-covalent association) and thus result mostly in FNs. “Convoluted text excerpt” refers to text segments characterized by syntactic intricacies, including complex sentences and cross-sentence relationships, which are inherently difficult to understand, in some cases even for humans. A separate error type related to this is “co-reference resolution”. These errors arise when it is unclear, based on syntax, which subject(s) a specific Complex_formation relationship corresponds to, and produce FPs as well as FNs.

Finally, “annotation error” corresponds to cases where the annotators have made specific mistakes—often stemming from ambiguity—and these require fixing upon clearer examination. Recalculation of the statistics after excluding annotation errors lead to an increase in precision (89.7%), recall (80.1%), and F1-score (84.7%), representing a slight improvement compared to the original observations on the test set.

### 3.3 Trigger detection

#### 3.3.1 System evaluation

For large-scale relation extraction, we had trained a TensorFlow-based model that achieved 88.5% precision, 82.8% recall, and 85.5% F1-score on the ComplexTome development set. Since the Captum implementation of the LIG method operates only on models that are trained with the PyTorch library, we trained another relation extraction system (with the same RoBERTa-large-PM-M3-Voc encoder and the same best hyper-parameters) using the PyTorch library and during the implementation of the code, we also fixed a couple of minor implementation errors. This resulted in a better relation extraction model with 88.8% precision, 85.4% recall, and 87.1% F1-score on the development set. We used this model for developing and optimizing our trigger detection system, and also for large-scale execution of the trigger detection system on biomedical literature.

Trigger detection methods are usually evaluated with the standard metrics used for NER tasks (precision, recall, and F1-score). However, annotation of the trigger development set showed that (1) a trigger can span multiple input tokens, and (2) there can be more than one alternative trigger spans that are equally valid to annotate for a Complex_formation relationship (for more details, refer to our annotation guidelines). However, from the practical application standpoint, it is good enough that we recognize only a part of a multi-token trigger. Similarly, if there are alternative trigger spans for the same Complex_formation relationship (e.g. The CD40*-*TRAF2 *interaction*), recognizing one of them is sufficient. Therefore, as evaluation metrics for method development and optimization, we take average of precision scores, average of recall scores, and average of F1 scores for left-bound, right-bound, overlap and exact matching of detected trigger spans against the gold-standard annotations. These measures penalize the method when it fails to detect alternative trigger spans for a single Complex_formation relationship, but since they were easy to program and calculate, we used them for method development. For the final evaluation of our best method, we used manual evaluation, not penalizing for such cases.

For the development and optimization of the trigger detection system, we evaluated our methods on the positive Protein-Protein pairs in trigger development set. An initial experiment with the LIG- and SHAP-based methods showed that when the relation extraction model makes a mistake and produces a negative label for a positive pair (i.e. the FN predictions of the relation extraction system), any effort in detecting the trigger by model explanation methods leads to mistakes, producing incorrect triggers (FP trigger spans). For example, the SHAP method (with [CLS] and [SEP] tokens, and without the postprocessing heuristics) results in 62.1% overlap precision, 55.5% overlap recall, 58.6% overlap F1-score, but if we first discard all FN pairs, then the same method results in 67.7% precision, 52.7% recall, and 59.3% F1-score. From the perspective of end-users, having a higher precision is very important, because it will increase the credibility of text mining results. Therefore, for LIG- and SHAP-based methods, we chose to always check the relation label as predicted by the trained RE model, and only pursue trigger detection if a positive label has been predicted by the model. Table 4 shows the results of our best approaches on the trigger development set, before and after applying post-processing heuristics.

**Table 4.** Comparison of trigger detection methods on the trigger development set.^a^

Trigger detection method	Overlap Pre	Overlap Rec	Overlap F1	Exact Pre	Exact Rec	Exact F1	avg Pre	avg Rec	avg F1
Baseline	50.1	65.2	56.7	48.3	62.7	54.6	49.2	63.9	55.6
LIG (best layer = 9)	58.4	49.7	53.7	53.0	45.2	48.8	55.7	47.4	51.2
SHAP (with *[CLS]* and *[SEP]*)	62.2	55.8	58.8	56.9	51.5	54.1	59.5	53.8	56.5
SHAP (without *[CLS]* and *[SEP]*)	62.7	56.1	59.2	58.6	52.4	55.4	60.7	54.2	57.3
SHAP + heuristics (without *[CLS]* and *[SEP]*)	83.1	62.4	71.3	73.9	55.8	63.6	78.4	59.1	67.4
SHAP + heuristics (with *[CLS]* and *[SEP]*)	85.2	63.6	72.9	74.8	56.7	64.5	79.9	60.3	68.7
LIG (best layer = 14) + heuristics	95.1	70.9	81.3	85.8	63.9	73.3	90.5	67.4	77.3

a The best method (highlighted in gray, with avg F1 = 77.3%) is used for large-scale execution on the entire literature. Precision, recall, and F1-score values are presented in percentages (%).

Pre, precision; Rec, recall; F1, F1-score; avg, average of left-bound, right-bound, overlap, and exact matching scores.

As shown in Table 4, the baseline method performs very poorly, achieving 55.6% average F1-score, showing that a simple pattern matching approach is not good for trigger detection. This is mostly due to the high amount of FP spans that resulted in the lowest average precision among all methods.

Another interesting observation is that the LIG method (without postprocessing heuristics) performs the worst. Although having a higher precision than the baseline, this method yields a much lower recall, and lower F1-score, suggesting that the LIG method without additional heuristics is not fit for the job. In contrast, the two SHAP-based methods (without the heuristics) have performed closely, with 56.5% and 57.3% F1-score, and slightly outperformed the baseline, showing that “right out of the box” and without additional tweaking, vanilla SHAP methods outperform the LIG method on the task.

The postprocessing heuristic rules significantly improve the results, increasing both precision and recall, which is evident in both SHAP- and LIG-based methods. For example, the SHAP method (without the [CLS] and [SEP] tokens) has achieved 67.4% avg. F1-score with the heuristics, outperforming the vanilla SHAP method by ∼10%. Similarly, the SHAP (with [CLS] and [SEP] tokens) achieved 68.7% F1-score with the heuristics, ∼12% higher than the vanilla SHAP method. Finally, our best results have been achieved by using the vectors obtained from the LIG method for the 14th hidden layer in the neural network model and applying the postprocessing rules. This method resulted in the highest precision, highest recall and F1-score (overlap, exact matching, and average). For example, the 85.8% exact precision shows that in ∼86% of the cases, detected spans are the actual triggers, and the 95.1% overlap precision reflects that in ∼95% of the cases, detected spans overlap with the actual trigger spans, which is sufficient for the application point of view, because for large-scale execution, we prefer not to mark any spans if the method is not sure, but we want to make sure the detected spans overlap with the actual trigger spans. This method achieved 90.5% average precision, 67.4% average recall, and 77.3% average F1-score, and it is used for large-scale trigger detection for STRING v12. These calculations penalize all methods in cases where multiple trigger spans are annotated for a relation, resulting to significantly lower recall. For that reason, we manually calculated the overlap performance metrics of the best method (LIG+Heuristics) *without penalizing* for alternate trigger spans, to get an accurate picture of the performance on the development set and this resulted in a precision of 95.9%, a recall of 83.1% (increase by ∼12%), and an F1-score of 89.1%.

#### 3.3.2 Manual error analysis

Manual recalculation of the overlap performance metrics on the trigger test set yielded 92.8% precision, 79.9% recall, and 85.9% F1-score. Detailed results are provided in Supplementary Material Section S8. A closer look in the results produced by the method shows that in cases where multiple words are valid as triggers, the method has a preference for punctuation (i.e. “-” or “/” in 35 cases) instead of whole words (e.g. “complex” in five cases). In cases where the method misdetects a trigger, by missing to predict the annotated trigger producing an FN, and by producing an incorrect trigger, i.e. an FP, there is no special pattern in the generated FPs, and the most frequent FN is the word “complex”. FN Complex_formation relation predictions of the RE system, for which we had chosen not to run the trigger detection method as discussed above, inevitably result to FN predictions for the trigger detection method as well. Manual evaluation did not show any specific patterns pertaining these FNs. We also detected four annotation errors, where multiple triggers where valid and not all of them were annotated. This has been taken into consideration during the calculation of performance metrics above.

### 3.4 Large-scale execution and integration in STRING v12

To perform relation extraction and trigger detection for STRING v12, we used all PubMed abstracts (as of August 2022) and all full texts available in the PMC BioC text mining collection (Comeau *et al.* 2019; as of April 2022). The entire corpus consists of 34 420 049 documents. We converted all documents into BRAT standoff format and obtained NER and NEN Protein annotations from Jensenlab tagger (Jensen 2016) for both abstracts and full-text articles. 6 033 981 documents (3 604 037 abstracts and 2 429 944 full-texts) contained at least two protein NEs and were provided for Complex_formation relation prediction to the model with the best performance on the RE task. We selected documents containing at least two NEs as this is a prerequisite for having relations. There was no upper limit on the number of NEs in the documents, as our RE system processes entities one pair at a time, with no restrictions on the number of pairs processed, apart from the maximum sequence length, as explained in Section 2. This resulted in predictions for over 1 billion NE pairs. From those 8 807 592 (<1%) are predicted as Complex_formation relationships. We then provide the ∼8.8 million “positive” examples as input for the trigger detection pipeline. 7 127 119 of those examples have at least one trigger predicted. A tab-delimited file with scores produced by our RE model for all NE pairs, and the BRAT-formatted input and tab-delimited results from large-scale execution of the trigger detection system are provided through Zenodo.

On top of our gold-standard evaluation against the ComplexTome test set, we evaluated the performance of the best RE model on large-scale extraction by selecting 1000 random documents from the 34 420 049 papers in the scientific literature and assessing the acceptability of extractions the system made. Of the 1000 random documents, 188 mention at least two proteins, and 31 contain at least one positive prediction. We manually evaluated these predictions and found 156 TPs, 9 FPs, and 23 FNs in these documents, leading to a precision = 94.5%, a recall = 87.2%, and an *F*-score = 90.7%. These results confirm the quality of the predictions produced by the best RE model, on a completely unbiased selection of documents. The fact that these results are better than those we had on the test set is probably explained by the fact that this type of assessment measures the acceptability of extractions rather than whether they would have been annotated by a human given the text without annotations. The former is arguably a looser criterion compared to evaluation against a gold standard. Detailed results for this analysis and BRAT-formatted files for manual inspection are provided via Zenodo.

Starting from version 11.5 of the STRING database (Szklarczyk *et al.* 2021), users gained access to a separate mode that provides a physical interaction subnetwork besides the broader functional association STRING network. Herein we have described a complete re-implementation of the text-mining pipeline that allows the detection of Complex_formation relationships (equivalent to “physical interactions” in STRING). The results from the large-scale run are properly processed and incorporated in STRING. For details on post-processing please refer to the STRING publications (Szklarczyk *et al.* 2021, 2023). In the physical interaction subnetwork of STRING v11.5, if there was evidence of a physical interaction between two unique Protein NEs based on text mining, the web interface enabled users to explore the publications supporting this interaction. This was accomplished by presenting actual text excerpts from biomedical articles, with the recognized Protein NEs highlighted. This feature served a dual purpose: users could personally evaluate the accuracy of automatically extracted relations, and they could also refer to the original articles for further in-depth reading.

Recognizing the potential for even greater utility, in the current version of STRING (Szklarczyk *et al.* 2023) it became desirable to have the system highlight the most significant word or phrase in the text snippet that explicitly or implicitly indicates the relation for each physical interaction extracted from that snippet. To achieve this, the results from the large-scale run of the trigger detection system were utilized. Supplementary Material Section S9 shows how text-mining results are presented for the physical interaction between two proteins in STRING v11.5 and v12.

## 4 Conclusions

In this work, we present both ComplexTome, a corpus tailored for relation extraction of complex formation relationships among biomedical entities, and a relation extraction system that allows supervised training on the task, alongside large-scale execution on the biomedical literature. On top of relation extraction we also present a trigger detection method for Complex_formation relationships. Both the relation extraction and trigger detection methods achieve high levels of accuracy (*F*-score = 82.8% and 85.9% on the test set, respectively) and are available for use by the entire scientific community. We have meticulously manually evaluated both systems and integrated them into the latest version of the STRING database. As a result, we have not only augmented the database’s coverage of physical interactions but also empowered users to explore and validate these relationships in the context of the original scientific articles. Overall, this project exemplifies the continuous evolution of text-mining capabilities in the era of language modeling and marks a significant milestone in enhancing our understanding of complex biological systems within the biomedical domain.

## Supplementary Material

btae552_Supplementary_Data

## Data Availability

All resources introduced in this study are available under open licenses through Zenodo (https://doi.org/10.5281/zenodo.8139716) and GitHub (https://github.com/farmeh/ComplexTome_extraction).

## References

[btae552-B1] Björne J , Heimonen J, Ginter F et al Extracting complex biological events with rich graph-based feature sets. In: *Proceedings of the BioNLP 2009 Workshop Companion Volume for Shared Task*, Boulder, CO: Association for Computational Linguistics, 2009, 10–18.

[btae552-B2] Bunescu R , Ge R, Kate RJ et al Comparative experiments on learning information extractors for proteins and their interactions. Artif Intell Med 2005;33:139–55. 15811782 10.1016/j.artmed.2004.07.016

[btae552-B3] Chen Q , Sun H, Liu H et al An extensive benchmark study on biomedical text generation and mining with chatgpt. Bioinformatics 2023;39:btad557. 37682111 10.1093/bioinformatics/btad557 PMC10562950

[btae552-B4] Cohen J. A coefficient of agreement for nominal scales. Educ Psychol Meas 1960;20:37–46.

[btae552-B5] Comeau DC , Wei C-H, Islamaj Doğan R et al Pmc text mining subset in bioc: about three million full-text articles and growing. Bioinformatics 2019;35:3533–5. 30715220 10.1093/bioinformatics/btz070 PMC6748740

[btae552-B6] Devlin J , Chang M-W, Lee K et al Bert: Pre-training of deep bidirectional transformers for language understanding. In: *Proceedings of NAACL-HLT*, Vol. 1, Minneapolis, MN: Association for Computational Linguistics, 2019, 4171–86.

[btae552-B7] Dewi C , Tsai B-J, Chen R-C. Shapley additive explanations for text classification and sentiment analysis of internet movie database. In: Szczerbicki E, Wojtkiewicz K, Nguyen SV, Pietranik M, Krótkiewicz M (eds.), Recent Challenges in Intelligent Information and Database Systems. Singapore: Springer Nature Singapore, 2022, 69–80. ISBN 978-981-19-8234-7.

[btae552-B8] Franceschini A , Szklarczyk D, Frankild S et al String v9. 1: protein-protein interaction networks, with increased coverage and integration. Nucleic Acids Res 2012;41:D808–15. 23203871 10.1093/nar/gks1094 PMC3531103

[btae552-B9] Fundel K , Küffner R, Zimmer R. RelEx—relation extraction using dependency parse trees. Bioinformatics 2006;23:365–71. 10.1093/bioinformatics/btl616 17142812

[btae552-B10] Gillespie M , Jassal B, Stephan R et al The reactome pathway knowledgebase 2022. Nucleic Acids Res 2022;50:D687–92. 34788843 10.1093/nar/gkab1028 PMC8689983

[btae552-B11] Giurgiu M , Reinhard J, Brauner B et al Corum: the comprehensive resource of mammalian protein complexes—2019. Nucleic Acids Res 2019;47:D559–63. 30357367 10.1093/nar/gky973 PMC6323970

[btae552-B12] Jahan I , Laskar MTR, Peng C et al Evaluation of chatgpt on biomedical tasks: a zero-shot comparison with fine-tuned generative transformers. In: *Workshop on Biomedical Natural Language Processing*, Toronto, Canada: Association for Computational Linguistics, 2023. https://api.semanticscholar.org/CorpusID:259096053.

[btae552-B13] Jensen LJ. One tagger, many uses: Illustrating the power of ontologies in dictionary-based named entity recognition. In: *Proceedings of the Joint International Conference on Biological Ontology and BioCreative*, Corvallis, OR: CEUR-WS.org, 2016.

[btae552-B14] Jimenez Gutierrez B , McNeal N, Washington C et al Thinking about GPT-3 in-context learning for biomedical IE? think again. In: *Findings of the Association for Computational Linguistics: EMNLP 2022*. Abu Dhabi, United Arab Emirates: Association for Computational Linguistics, 2022, 4497–512. 10.18653/v1/2022.findings-emnlp.329

[btae552-B15] Kim CY , Baek S, Cha J et al HumanNet v3: an improved database of human gene networks for disease research. Nucleic Acids Res 2021;50:D632–9. 10.1093/nar/gkab1048. PMC8728227 34747468

[btae552-B16] Kim J-D , Ohta T, Tsujii J. Corpus annotation for mining biomedical events from literature. BMC Bioinformatics 2008; 9:10. https://api.semanticscholar.org/CorpusID:5261517. 18182099 10.1186/1471-2105-9-10 PMC2267702

[btae552-B17] Kim J-D , Ohta T, Pyysalo S et al Overview of BioNLP’09 shared task on event extraction. In: *Proceedings of the BioNLP 2009 Workshop Companion Volume for Shared Task*. Boulder, Colorado: Association for Computational Linguistics, 2009, 1–9. https://aclanthology.org/W09-1401.

[btae552-B18] Krallinger M , Leitner F, Rodriguez-Penagos C et al Overview of the protein-protein interaction annotation extraction task of biocreative II. Genome Biol 2008;9 Suppl 2:S4–19. 10.1186/gb-2008-9-s2-s4 PMC2559988 18834495

[btae552-B19] Lee J , Yoon W, Kim S et al BioBERT: a pre-trained biomedical language representation model for biomedical text mining. Bioinformatics 2019;36:1234–40. 10.1093/bioinformatics/btz682 PMC7703786 31501885

[btae552-B20] Lewis P , Ott M, Du J et al Pretrained language models for biomedical and clinical tasks: Understanding and extending the state-of-the-art. In: *Proceedings of the 3rd Clinical Natural Language Processing Workshop*. Association for Computational Linguistics. 10.18653/v1/2020.clinicalnlp-1.17, Online, November 2020, 146–57.

[btae552-B21] Licata L , Briganti L, Peluso D et al Mint, the molecular interaction database: 2012 update. Nucleic Acids Res 2012;40:D857–61. 22096227 10.1093/nar/gkr930 PMC3244991

[btae552-B22] Lundberg SM , Lee S-I. A unified approach to interpreting model predictions. Paper presented at 31st Conference on Neural Information Processing Systems (NIPS 2017). 2017. https://github.com/slundberg/shap

[btae552-B23] McHugh ML. Interrater reliability: the kappa statistic. Biochem Med (Zagreb) 2012;22:276–82. 23092060 PMC3900052

[btae552-B24] Mehryary F , Björne J, Pyysalo S et al Deep learning with minimal training data: TurkuNLP entry in the BioNLP shared task 2016. In: *Proceedings of the 4th BioNLP Shared Task Workshop*, Berlin, Germany. Association for Computational Linguistics, 2016, 73–81. 10.18653/v1/W16-3009.

[btae552-B25] Mehryary F , Björne J, Salakoski T et al Potent pairing: ensemble of long short-term memory networks and support vector machine for chemical-protein relation extraction. Database 2018;2018:bay120. 30576487 10.1093/database/bay120 PMC6310522

[btae552-B26] Mehryary F , Moen H, Salakoski T et al Entity-pair embeddings for improving relation extraction in the biomedical domain. In: *28th European Symposium on Artificial Neural Networks, Computational Intelligence and Machine Learning, ESANN 2020 (online event)*. i6doc.com publication, 2020, 613–8. ISBN 978-2-87587-073-5.

[btae552-B27] Meldal BHM , Bye-A-Jee H, Gajdoš L et al Complex portal 2018: extended content and enhanced visualization tools for macromolecular complexes. Nucleic Acids Res 2019;47:D550–8. 30357405 10.1093/nar/gky1001 PMC6323931

[btae552-B28] Milošević N , Thielemann W. Comparison of biomedical relationship extraction methods and models for knowledge graph creation. J Web Semantics 2023;75:100756. 10.1016/j.websem.2022.100756

[btae552-B29] Miranda-Escalada A , Mehryary F, Luoma J et al Overview of drugprot task at biocreative vii: Data and methods for large-scale text mining and knowledge graph generation of heterogenous chemical–protein relations. Database 2023;2023:baad080. 38015956 10.1093/database/baad080 PMC10683943

[btae552-B30] Nédellec C. Learning language in logic—genic interaction extraction challenge. In: *Learning Language in Logic Workshop (LLL05)*, Born, Germany: ACM-Association for Computing Machinery, 2005.

[btae552-B31] OpenAI J , Achiam S, Adler S et al Gpt-4 technical report. arXiv, arXiv:2303.08774v6, 2024, preprint: not peer reviewed.

[btae552-B32] Orchard S , Ammari M, Aranda B et al The mintact project—intact as a common curation platform for 11 molecular interaction databases. Nucleic Acids Res 2014;42:D358–63. 24234451 10.1093/nar/gkt1115 PMC3965093

[btae552-B33] Oughtred R , Rust J, Chang C et al The biogrid database: a comprehensive biomedical resource of curated protein, genetic, and chemical interactions. Protein Sci 2021;30:187–200. 33070389 10.1002/pro.3978 PMC7737760

[btae552-B34] Pyysalo S , Ginter F, Heimonen J et al Bioinfer: a corpus for information extraction in the biomedical domain. BMC Bioinformatics 2007;8:50–24. 17291334 10.1186/1471-2105-8-50 PMC1808065

[btae552-B35] Pyysalo S , Airola A, Heimonen J et al Comparative analysis of five protein-protein interaction corpora. *BMC Bioinformatics* 2008;**9**(Suppl 3):S61–11. 10.1186/1471-2105-9-S3-S6 PMC2349296 18426551

[btae552-B36] Pyysalo S , Ohta T, Tsujii J. Overview of the entity relations (rel) supporting task of bionlp shared task 2011. In: Tsujii J, Kim J-D, Pyysalo S (eds.), *BioNLP@ACL*. Association for Computational Linguistics, 2011, 83–88. https://api.semanticscholar.org/CorpusID:12635424.

[btae552-B37] Stenetorp P , Pyysalo S, Topić G et al brat: a web-based tool for NLP-assisted text annotation. In: *Proceedings of the Demonstrations at the 13th Conference of the European Chapter of the Association for Computational Linguistics*. Avignon, France: Association for Computational Linguistics, 2012, 102–107. https://aclanthology.org/E12-2021.

[btae552-B38] Sundararajan M , Taly A, Yan Q. Axiomatic attribution for deep networks. In: *Proceedings of the 34th International Conference on Machine Learning—Volume 70, ICML’17*, Sydney, NSW, Australia: JMLR.org, 2017, 3319–28.

[btae552-B39] Szklarczyk D , Gable AL, Nastou KC et al The string database in 2021: Customizable protein–protein networks, and functional characterization of user-uploaded gene/measurement sets. Nucleic Acids Res 2021;49:D605–12. 33237311 10.1093/nar/gkaa1074 PMC7779004

[btae552-B40] Szklarczyk D , Kirsch R, Koutrouli M et al The string database in 2023: Protein–protein association networks and functional enrichment analyses for any sequenced genome of interest. Nucleic Acids Res 2023;51:D638–46. 36370105 10.1093/nar/gkac1000 PMC9825434

[btae552-B41] Touvron H , Martin L, Stone K et al Llama 2: open foundation and fine-tuned chat models. arXiv, arXiv:2307.09288 2023, preprint: not peer reviewed.

[btae552-B42] Vaswani A , Shazeer N, Parmar N et al Attention is all you need. In: *Proceedings of the 31st International Conference on Neural Information Processing Systems, NIPS’17*, Red Hook, NY, USA: Curran Associates Inc., 2017, 6000–6010 ISBN 9781510860964.

[btae552-B43] Wan Z , Cheng F, Mao Z et al GPT-RE: In-Context Learning for Relation Extraction Using Large Language Models. In: *Proceedings of the 2023 Conference on Empirical Methods in Natural Language Processing*, Singapore: Association for Computational Linguistics, 2023, 3534–3547.

[btae552-B44] Wang S , Sun X, Li X et al Gpt-NER: Named Entity Recognition Via Large Language Models. arXiv, arXiv:2304.10428, 2023, preprint: not peer reviewed.

