# Supplementary material for: STRING-ing together protein complexes: corpus and methods for extracting physical protein interactions from the biomedical literature
Source: Bioinformatics. 2024 Sep 14;40(9):btae552. doi: 10.1093/bioinformatics/btae552 (PMC11441320; doi:10.1093/bioinformatics/btae552)
Supplement: btae552_Supplementary_Data [file btae552_supplementary_data.pdf]

Supplementary material

“*STRING-ing together protein complexes: corpus and methods for extracting physical protein interactions from the biomedical literature*”

Farrokh Mehryary[1,†], Katerina Nastou[2,†], Tomoko Ohta[3], Lars Juhl Jensen[2] and Sampo Pyysalo[1]

[1]TurkuNLP Group, Department of Computing, University of Turku, Turku, Finland, [2]Novo Nordisk Foundation Center for Protein Research, University of Copenhagen, Blegdamsvej 3, 2200, Copenhagen, Denmark and [3]Textimi, Tokyo, Japan

[†] Equal contribution

1. Comparison of annotations in the BioNLP ST 2009 training and development datasets with ComplexTome annotations

To investigate whether currently available corpora can be used without reannotation for the purposes of training a relation extraction system for our downstream task, we binarized the event annotations from BioNLP ST 2009 training and development datasets into relation annotations and filtered them to binding relations only using the code available in this repository: https://github.com/spyysalo/binarize-events/. Then we compare these with the annotated relations in ComplexTome using the overlapping matching criterion, with the evaluation script available through the main repository for this project: https://github.com/farmeh/ComplexTome_extraction/blob/main/TrainRelationExtractionSystem/evalsorel.py

This comparison produced the following results for the overlapping set of documents in the two corpora:

- **209 relations in common** between the BioNLP ST 2009 sets and ComplexTome
- **137 relations** annotated only in ComplexTome
- **108 relations** annotated only in BioNLP ST 2009 sets

In summary there is less than 50% overlap between the relation annotations of these two datasets. This result can easily be explained by the different definition of binding in the two sets, as well as differences in named entity annotation as discussed in the Introduction section of our manuscript. This experiment shows that using the annotations from this dataset for transfer learning would not have worked as intended and showcases that reannotating the documents was the correct decision.

2. Implementation of marking and masking approach in the relation extraction system

Since a `Complex_formation` relation by definition is non-directional (i.e., `R(e1,e2)=R(e2,e1)`), an input document with N NEs includes $N!/((N-2)!\times 2)$ candidate entity pairs, and for each pair, the system has to predict and assign a positive or a negative label. A typical document usually contains more than two entities (i.e., there are more than one pair in a typical document), therefore it is necessary to inform the classifier which two particular NEs constitute a pair at-a-time for label prediction. For this aim, we transform the text by encoding the entities in the input document, either using a marking approach or a masking approach. We use language model's "`unused`" tokens for this aim.

The marking approach:

In our marking approach, only the two focused entities (that constitute a pair at-a-time) are marked, but the texts of other entities in the text remain untouched. We use different "unused" tokens to mark the beginning and end of the focused entities, based on their type (e.g., `[unused1]` and `[unused2]` tokens are used to mark a `Protein` entity boundary, `[unused3]` and `[unused4]` are used to mark a `Chemical` boundary, etc). Therefore, our marking approach not only denotes which two entities constitute a pair for label detection, but it also denotes their type, providing maximum information to the classifier.

Following is an example sentence with three `Protein` entities and a `Protein_Family` entity and shows how the marking approach works. Note how different "unused" tokens are utilized to denote entity boundaries based on entity type.

Sentence:

"**GrpL**, a **Grb2-related adaptor protein**, interacts with **SLP-76** to regulate **nuclear factor of activated T cell** activation."

Entities:

`Protein` entities = {"**GrpL**", "**Grb2-related adaptor protein**", "**SLP-76**"}
`Protein_Family` entities = {"**nuclear factor of activated T cell**"}

There are six candidate pairs in the sentence which are transformed differently based on the focused entities:

- "[unused1]**GrpL**[unused2], a [unused1]**Grb2-related adaptor protein**[unused2], interacts with SLP-76 to regulate nuclear factor of activated T cell activation."

- "[unused1]**GrpL**[unused2], a Grb2-related adaptor protein, interacts with [unused1]**SLP-76**[unused2] to regulate nuclear factor of activated T cell activation."

- "[unused1]**GrpL**[unused2], a Grb2-related adaptor protein, interacts with SLP-76 to regulate [unused7]**nuclear factor of activated T cell**[unused8] activation."

- "GrpL, a [unused1]**Grb2-related adaptor protein**[unused2], interacts with [unused1]**SLP-76**[unused2] to regulate nuclear factor of activated T cell activation."

- "GrpL, a [unused1]**Grb2-related adaptor protein**[unused2], interacts with SLP-76 to regulate [unused7]**nuclear factor of activated T cell**[unused8] activation."

- "GrpL, a Grb2-related adaptor protein, interacts with [unused1]**SLP-76**[unused2] to regulate [unused7]**nuclear factor of activated T cell**[unused8] activation."

The masking approach

In the masking approach, the texts of the two focused entities in the text are always masked with [unused1] tokens, while **all other entities** in the text are masked with [unused2] tokens. Therefore, not only we hide the texts of all entities, but we also hide their types across the whole document. This is to ensure maximum generalization on unseen texts as the neural network model has to rely on and learn from non-entity words as context.

Following are the 6 transformations of the mentioned sentence, based on the masking approach:

- "**[unused1]**, a **[unused1]**, interacts with [unused2] to regulate [unused2] activation."
- "**[unused1]**, a [unused2], interacts with **[unused1]** to regulate [unused2] activation."
- "**[unused1]**, a [unused2], interacts with [unused2] to regulate **[unused1]** activation."

- "[unused2], a **[unused1]**, interacts with **[unused1]** to regulate [unused2] activation."
- "[unused2], a **[unused1]**, interacts with [unused2] to regulate **[unused1]** activation."
- "[unused2], a [unused2], interacts with **[unused1]** to regulate **[unused1]** activation."

A note on example generation:

For each candidate named-entity pair, after marking/masking the entities in the input text, we tokenize the transformed text into its corresponding sub-tokens (based on the vocabulary of a particular language model currently being used) and for each pair, we calculate their distance in sub-tokens (including the added markers in case of marking) and if they can fit into a window with a size smaller than or equal to the specified **MSL**, we generate a machine learning example (a sequence of tokens as a neural network input) for the pair.

We highlight that the same example generation method is used for positive/negative examples (i.e., during the training), and for unlabeled examples (i.e., during the prediction). Those longer examples not fitting into a window will be either discarded (in the case of training or predicting the unlabeled examples in large-scale prediction) or will be counted as False Negative (FN) predictions of the system (if there is a Complex_formation relation between their entities, in case of development/test set pairs).

3. Relation extraction system details

3.1 System architecture and the transformer-based models

For building the relation extraction system, we follow the mainstream and common approach of fine-tuning a pre-trained transformer-based model on the ComplexTome training set for binary relation extraction. Therefore, the relation extraction system is based on deep neural networks and its architecture is composed of one pre-trained transformer encoder, followed by a decision layer with a softmax activation function, for binary classification.

The current state-of-the-art text mining methods in the biomedical domain utilize models based on the transformer architecture [1], and for that reason, we have also focused our efforts on these. In particular, recent studies have shown that the RoBERTa-large-PM-M3-Voc model [2] has resulted in the highest performance scores in several biomedical text mining tasks. For example, in the Drug-Protein relation extraction task of the BioCreative VII challenge [3], the winning team obtained an F1 score of 79.73% on the hidden test set using this model and achieved the first rank out of 107 submitted runs in the official evaluation [4]. In a similar study, Luoma et al. (2023) have developed a transformer-based system for biomedical Named-Entity Recognition (NER) and compared three language models that are specifically pre-trained for the biomedical domain (RoBERTa-large-PM-M3-Voc, BioBERT-large, cased [6], and BioMegatron 345M Bio-vocab-50k [7]), and they have shown that the RoBERTa-large-PM-M3-Voc model outperforms the rest [5].

Since we are using the Hugging Face transformers library, it enables us to seamlessly plug and try different pre-trained transformer models (available in the model repository) into the neural network architecture and fine-tune it on the ComplexTome training set (for details on example generation and how texts of candidate named-entity pairs are fed to the neural network model, see Section 2). When doing so, we make sure to do a full grid search to find the optimal values of hyper-parameters (including the learning rate, number of training epochs, mini-batch size, and max sequence length), and to deal with the effect of initial random weights in the architecture on the performance metric (as measured on the development set), for each unique set of hyper-parameter values, we repeat each experiment for 4 times (training the network with the same hyper-parameters but with different initial random weights), and take the average and standard deviation of the resulting f1-scores.

We highlight that one round of grid search (and running 4 experiments per hyper-parameter set) on the GPU-cluster machine available to us takes

approximately one week to complete (varying based on the jobs already submitted to the queue by other research groups), therefore, we could not afford to try all transformer-based models that are pre-trained for the biomedical domain, and **chose to focus on the two most promising ones, BioBERT-large and RoBERTa-large-PM-M3-Voc,** based on the recent publications in the literature. These two models are tried for building the final system. However, initial experiments showed that RoBERTa-large-PM-M3-Voc outperforms the BioBERT-large model, therefore, it was used for building the final system. For the quick comparison of different training schemes (see here), we used the BioBERT-base model [6], since it is a smaller (yet very capable model), requiring much less training time. Finally, for the baseline system we used BERT-base-cased model [9].

3.1.1 RoBERTa and BERT architecture

The RoBERTa-large-PM-M3-Voc model is based on RoBERTa (Robustly Optimized BERT Approach) architecture [8], and it is trained on PubMed abstracts, PubMed Central full-text articles, the Medical Information Mart for Intensive Care, third update (MIMIC-III) texts, and other biomedical texts [2].

Both BERT and RoBERTa architectures are based on the Transformer architecture [1], using a multi-layer bidirectional Transformer encoder. The original Transformer model includes two main blocks: an encoder block and a decoder block. However, BERT and RoBERTa models are encoder-only models, meaning they only contain the encoder part (and not the decoder part), with a classifier added on top. The classifier predicts the masked tokens in the input (i.e. masked language modeling (MLM) task) and/or whether a second sentence precedes the first one or not (i.e. Next Sentence Prediction (NSP) task). Once pre-training a BERT or RoBERTa model is finished, it can be fine-tuned for different downstream tasks (e.g. relation extraction, question answering, NER, etc) with real training data, and in a supervised manner. Supplementary Figure 1 shows the general scheme for pre-training and fine-tuning a BERT model.

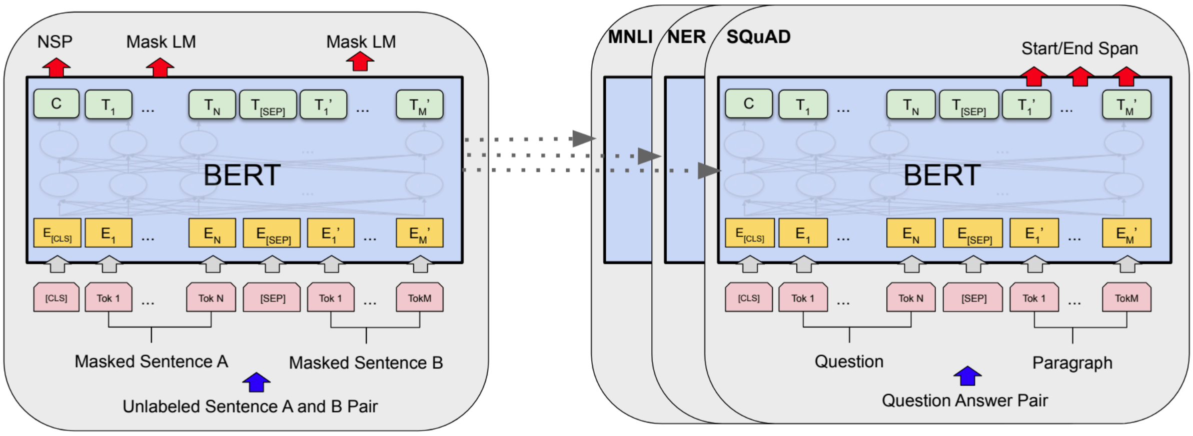

Pre-training Fine-Tuning

Supplementary Figure 1: Pre-training and fine-tuning a BERT model. The [CLS] token is a special token in the vocabulary and used to denote the start of a sequence (e.g. a sentence), and the [SEP] is a special separator token (e.g. separating questions/answers). Figure from Devlin et al. (2017).

In principle, RoBERTa models are very similar to the original BERT architecture [9] (both using the encoder block of the Transformer model [1]), but they utilize a few modifications in pre-training and architecture, which leads to improved performance. Here are the key differences between the two models:

1. The original BERT is pre-trained with two tasks: masked language modeling (MLM) and Next Sentence Prediction (NSP), whereas RoBERTA models are only trained with the MLM task because the authors have shown it results in better performance.

2. The original BERT was trained using static masking (which masks the *same* tokens at every epoch of pre-training), but RoBERTA models utilize dynamic masking, i.e. randomly masking different tokens at different points during pre-training, encouraging the model to learn more robust and generalizable representations of language by forcing it to predict missing tokens in a variety of different contexts.

3. RoBERTa models generally use byte-pair encoding (BPE) (a sub-word tokenization method that helps to handle rare and out-of-vocabulary words more effectively) with relatively larger vocabulary sizes in contrast to the original BERT model (that uses WordPiece sub-word tokenization with usually lower vocabulary size), resulting in better pre-trained models.

The aforementioned items are the main differences between RoBERTa and BERT models. However, in practice, other items (such as pre-training on longer texts, or pre-training with higher number of epochs) can be utilized when making a BERT or a RoBERTa model to achieve better models and higher performance on downstream tasks.

3.1.2 Transformer architecture and key concepts

As discussed before, the Transformer model includes two main blocks: an encoder block and a decoder block [1]. Here we briefly discuss the two blocks in the architecture.

In principle, the encoder maps an input sequence of symbol representations (x1, ..., xn) (i.e. *sub-tokens*) to a sequence of continuous representations z = (z1, ..., zn). Given z, the decoder then generates an output sequence (y1, ..., ym) of symbols, one element at a time. At each step the model is auto-regressive, consuming the previously **generated** symbols as **additional input** when generating the next. The

Transformer follows this overall architecture using **stacked self-attention** and **point-wise**, **fully connected layers** for **both** the encoder and decoder (see Supplementary Figure 2).

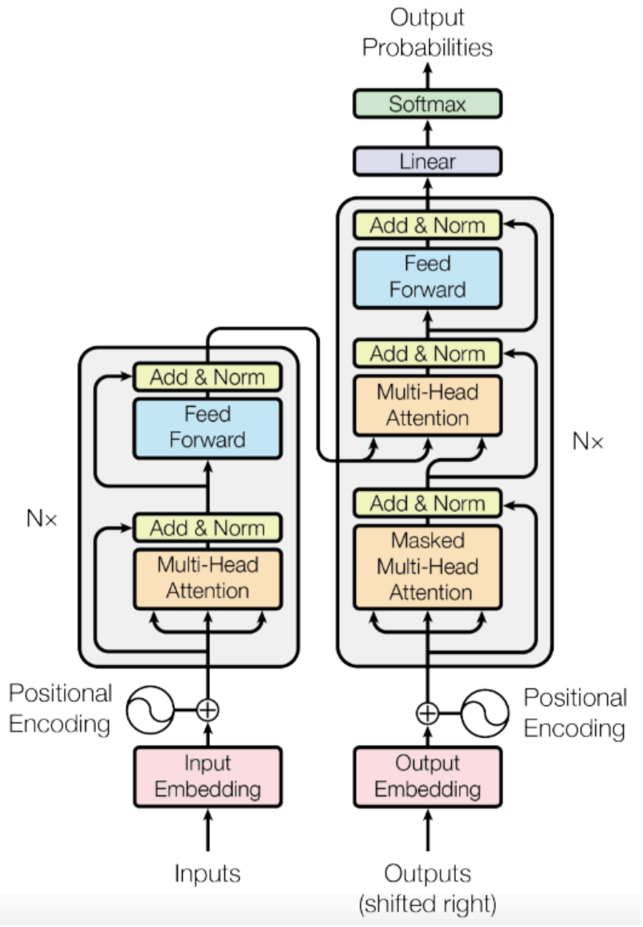

Supplementary Figure 2: Transformer model architecture. The left side shows the encoder block and the right side shows the decoder block. Each block is composed of N identical stacked layers. Figure adapted from Vaswani et al. (2017).

Encoder block: The encoder is composed of a stack of N identical layers. Each layer has two sub-layers. The first is a **multi-head self-attention mechanism**, and the second is a simple, position-wise fully connected feed-forward network. There is also a residual connection around each of the two sub-layers, followed by layer normalization. That is, the output of each sub-layer is LayerNorm(x + Sublayer(x)), where Sublayer(x) is the function implemented by the sub-layer itself. To facilitate these residual connections, all sub-layers in the model, as well as the embedding layers, produce the same outputs of dimension d_{model}. In both RoBERTa-large-PM-M3-Voc and BioBERT-large models that we have used, N=24.

Decoder block: The decoder is also composed of a stack of N identical layers. In addition to the two sub-layers in each encoder layer, the decoder inserts a third sub-layer, which performs multi-head attention over the **output of the encoder**

stack. Similar to the encoder, there are residual connections around each of the sub-layers, followed by layer normalization. The self-attention sub-layer in the decoder stack is also modified to prevent positions from attending to subsequent positions.

Attention mechanism in the encoder/Decoder:

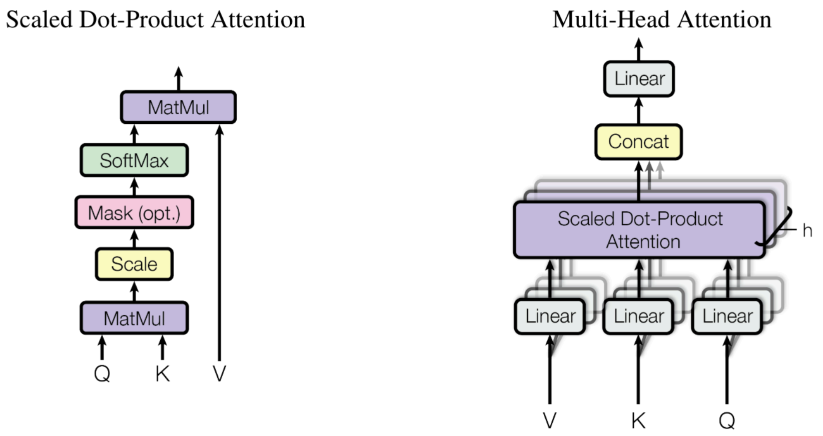

Supplementary Figure 3: (left) Scaled Dot-Product Attention. (right) Multi-Head Attention consists of several attention layers running in parallel. Figure adapted from Vaswani et al. (2017).

Each layer in the encoder and decoder is based on using the multi-head attention mechanism. An attention function can be described as the **mapping** of a query (Q) and a set of key-value pairs (K, V) to an output, where the query, keys, values, and output are all vectors. The output is computed as a weighted sum of the values, where the weight assigned to each value is computed by a compatibility function of the query with the corresponding key.

- **Scaled Dot-Product Attention (Supplementary Figure 3):** The particular attention mechanism implemented in the architecture is called "Scaled Dot-Product Attention" (see Supplementary Figure 3). The input consists of queries and keys of dimension d_k, and values of dimension d_v. Attention is calculated based on the following equation:

$$\mathrm{Attention}(Q, K, V) = \mathrm{softmax}(\frac{QK^T}{\sqrt{d_k}})V$$

- **Multi-Head Attention (Supplementary Figure 3):** Instead of using a single attention function, the architecture uses multiple attention functions, and they are aggregated based on the following equation:

$$\mathrm{MultiHead}(Q, K, V) = \mathrm{Concat}(\mathrm{head_1}, ..., \mathrm{head_h})W^O$$
$$\text{where } \mathrm{head_i} = \mathrm{Attention}(QW_i^Q, KW_i^K, VW_i^V)$$

Where the projections are parameter matrices $W_i^Q \in \mathbb{R}^{d_{\text{model}} \times d_k}$, $W_i^K \in \mathbb{R}^{d_{\text{model}} \times d_k}$, $W_i^V \in \mathbb{R}^{d_{\text{model}} \times d_v}$ and $W^O \in \mathbb{R}^{hd_v \times d_{\text{model}}}$.

Multi-head attention allows the model to jointly attend to information from different representation subspaces at different positions. With a single attention head, averaging inhibits this. Finally, we would like to refer the reader to The Illustrated Transformer web page, for further reading about the inner workings of the transformer architecture.

3.2 List of transformer models and hyper-parameters used for building the relation extraction system

Models	**BERT-base-cased** (download)(paper): used in the baseline system
	**BioBERT-base** (download) (paper): used for quick comparison of different training schemes.
	**BioBERT-large** (download) (paper): used for building the relation extraction system. This is a large model with 24 hidden layers in the transformer encoder.
	**RoBERTa-large-PM-M3-Voc** (download) (paper): used for building the relation extraction system. This is a large model with 24 hidden layers in the transformer encoder.
Max sequence length	128, 144, 160, 176, 192
Learning rate	2e-06, 3e-06, 4e-06, 5e-06
Mini-batch size	• 5 (for the large models and BERT-base-cased in the baseline system)<br>• 16 (for BERT-base-cased and BioBert-base model)
Number of epochs	6,7,8,9,10,11,12

We used the **BERT-base-cased** model for the baseline system, and **BioBERT-base** model for quick comparison of different training schemes (see here). For building the final system we tested BioBERT-large and RoBERTa-large-PM-M3-Voc models since these two models have recently achieved state-of-the-art on various tasks. Initial experiments showed that RoBERTa-large-PM-M3-Voc outperforms the BioBERT-large model, therefore, it was used for building the final system.

4. Different training schemes and evaluation results

The corpus contains **four** named-entity types (Protein, Chemical, Complex, Protein_Family/Family) and Complex_formation relationships can occur between any two entities mentioned in the text. For the real-world application for which the model was intended to be used (i.e., extracting Protein-Protein interactions for the STRING database v12), the system has to deal with texts including only Protein entities. Hence, to have a realistic optimization method for large-scale prediction, we filter out all non-Protein entities and all Protein entities with the "blocklisted" attribute (and their relations) from the development and the test sets. In order to remove the Protein entities with the “blocklisted” attribute, we first convert them into a new entity type (Protein_BL) to allow for their easy removal when necessary.

We performed experiments with five different training schemes detailed below. Supplementary Table 1 shows an overview of each training scheme along with the highest f1-score achieved on the development set.

We used the BioBert-base model for all of the above training schemes (since it is a relatively small language model, thus requiring less computational resources, and we can get the results faster). For each training scheme, we run a full grid search to find the optimal values of hyper-parameters. For each hyper-parameter set, we repeat each experiment four times and calculate the average and standard deviation of the f1-score. Finally, we find and report the best obtained f1-score average and standard deviation. Following is the explanation of each approach.

Training scheme 1-A: In this approach, we use all available training data (2489 annotated Complex_formation relations among all entity types), and use the marking approach to denote the candidate entities in the input texts and their types. As Table1 shows, this approach yields the highest f1-score with an average of 81.10%and 0.2915 standard deviation.

Training scheme 1-B: This approach is similar to **1-A**, however, instead of marking the candidate entities, we mask all entity texts and their types, resulting in an average f1-score of 80.20%.

Training scheme 2: In this approach, we filter out all (i.e., delete) annotations for non-Protein entities and any annotated Complex_formation between them from the training set, and use the masking approach to hide all remaining entities and their types. This approach results in the minimum number of training examples

(1961), but is the most similar set to the reduced development set, i.e., the set that only contains Protein entities and interactions among them. This approach resulted in 79.28% average f1-score with 0.36 standard deviation. Please note that annotations for Protein_BL, Complex, Chemical and Family entities are deleted from the training .ann files, hence their texts are not masked in the experiment, and constitute part of the contexts if they appear in the window of Protein-Protein training examples.

Training scheme 3: In this approach, we only use entities of types Protein, Complex and Protein_BL (by converting the type of all Complex and Protein_BL entities to Protein), and then use the masking approach to mask the texts of all entities and their types (similar to Training scheme 1-B). As a result, Family and Chemical entities in context are retained, but no training examples for candidate pairs that either one or both of the entities are Family or Chemical are generated. The reasoning behind this training scheme is that in the scientific literature people tend to discuss Complex and Protein_BL entities in a similar manner as Protein entities, so there could be a benefit from using all of them while training, since this allows for a higher support for Complex_formation relationships (2117).

Training scheme 4: This approach is very similar to Training scheme 3, in the sense that only entities of types Protein, Complex and Protein_BL are masked and used to generate examples. The difference in this case is that annotations for Family and Chemical entities are completely removed and thus the text of these entities now constitutes part of the contexts if it appears within the window of a training example. This experiment was done to explore whether the text of Family and/or Chemical entities is important for the model towards predicting a Complex_formation relationship between two entities.

Out of all four schemes we have chosen to use **training scheme 1-B** for our relation extraction model training. There are two main reasons for this choice. Firstly, in comparison to training schemes 2,3 and 4, where masking is used as an encoding type, there is no statistically significant difference to the results obtained on the development set and the fact that we have the maximum amount of training data and maximum masking in this case, means that this is the most generalizable approach, out of these 4. When it comes to comparing with training scheme 1-A, the only difference is the approach used for encoding types between the two experiments. Again the difference is not statistically significant (i.e. the difference of the mean F-scores is within ±3std) and the masking approach is more generalizable to the open-world scenario, thus we have chosen training scheme 1-B for all subsequent experiments.

Supplementary Table 1: Evaluation of different training schemes on the development set									
**Exp#**	**Encoding type**	**Training pairs (Positive/Negatives)**	**Masked entities**	**Not masked entities**	**Removed annotations**	**Comments**	**Highest mean(F1)**	**std(F1)**	**Positive pairs**
1-A	Marking	Pair = (e1, e2) e1, e2 ∈ {Protein, Protein_BL, Complex, Chemical, Family}		Protein, Protein_BL, Complex, Chemical, Family		Maximum training data<br>No masking	81.10	0.29	2489
1-B	Masking	Pair = (e1, e2) e1, e2 ∈ {Protein, Protein_BL, Complex, Chemical, Family}	Protein, Protein_BL, Complex, Chemical, Family			Maximum training data<br>Maximum masking	80.20	0.60	2489
2	Masking	Pair = (Protein, Protein)	Protein		Protein_BL, Complex, Chemical, Family	Minimum training data<br>Minimum masking<br>similar to filtered dev set	79.28	0.36	1961
3	Masking	Pair = (e1, e2) e1, e2 ∈ {Protein, Protein_BL, Complex}	Protein, Protein_BL, Complex, Chemical, Family			More training data compared to Experiment 2<br>Maximum masking	80.05	0.84	2117
4	Masking	Pair=(e1, e2) e1, e2 ∈ {Protein, Protein_BL, Complex}	Protein, Protein_BL, Complex,		Chemical, Family	More training data compared to Experiment 2<br>Less masking compared to Experiment 3	80.18	0.44	2117

5. Trigger Detection Methods

A. LIG-based trigger detection method

Our best relation extraction model was obtained by fine-tuning a pre-trained RoBERTa model (RoBERTa-large-PM-M3-Voc) on the relation extraction training set. We mainly focus on the outputs of the embedding layer and the outputs of the 24 hidden RoBERTa layers in this model. For each pair in the trigger development set, we feed the corresponding tokens as input to the model, and by applying the LIG method for all mentioned layers, we obtain 25 one-dimensional vectors, each representing the importance scores for the input tokens based on the outputs of a particular layer. By stacking the layers vertically, one can create a heatmap (rows denoting the layers, and columns denoting the tokens) and try to choose a layer (row in the heatmap) in which, the token with the highest score, is actually the trigger of the pair we are aiming to recognize.

The best layer might vary across different examples, thus we need a systematic approach for choosing the layer which yields the highest evaluation score when all predictions (i.e., predicted triggers for all positive pairs) are checked against the gold-standard (the annotated triggers in the trigger development set).

B. SHAP-based trigger detection method

Similarly to the LIG method, SHAP also yields a vector for the input tokens. For each pair, we simply feed the corresponding tokens as input to the model and apply the SHAP method, and then choose the token(s) with the highest score as the trigger(s). By repeating the process for all positive pairs, we obtain a prediction set which is then evaluated against the gold-standard. As an additional experiment, we try feeding the inputs *with* and *without* the [CLS] and [SEP] tokens, as initial experiments showed that the approaches produce slightly different results which demand further evaluation.

For both LIG and SHAP -based methods, we use the following approach: after obtaining a corresponding vector for a pair, we first discard the first and the last element of the vector (scores for the [CLS] and [SEP] tokens). Then we discard all “unused” tokens (which represent the entities) from the vector and then we choose the token(s) with the highest scores as the trigger(s) for that particular pair. By repeating the process for all positive pairs and for all layers, we obtain 25 LIG-based prediction sets (each based on choosing a particular layer as the best layer), and two SHAP-based prediction sets (with and without [CLS] and [SEP] tokens) and then we compare the prediction sets against the gold-standard and calculate evaluation scores.

C. Post-processing heuristic rules

Initial experiments showed that none of the two methods yield great evaluation scores, because sometimes tokens that are not actual triggers get the highest scores. To improve the results, we implemented the following post-processing heuristics. After a vector is calculated, and before selecting the token(s) with the highest score, we search all tokens in the vector and discard any token if one of the following rules applies:

- If the token is fully composed of white space(s) or punctuation mark(s) not found in the development set triggers ("-", ".","/" punctuation marks are actually valid triggers in the trigger development set).
- If \n or \t character is found inside the token text.
- If the token is a "." character and it is the first or the last token in the sequence (after removing [CLS] and [SEP] tokens)
- If the token is "." character, is located in the middle of the snippet, and it is not in the "[unused1].[unused1]" pattern.
- If the token belongs to a list of closed class words, such as pronouns, prepositions, conjugations, etc. The complete list of closed class words used is given in Supplementary Material Section 4E.

The aforementioned rules are obtained by inspecting the heatmaps and SHAP outputs for the (positive) pairs in the trigger development set. Finally, it is worth mentioning that our method can provide multiple disjoint trigger spans for a `Complex_formation` relationship, since we are finding the token(s) in a vector with the maximum score (i.e., if there are multiple tokens with the same highest score, they will all be returned as recognized triggers).

D. An example output for LIG and SHAP methods

The following shows the outputs of SHAP and part of the heatmap obtained by running layer integrated gradients with Captum. Please note that the two candidate named-entities are replaced with [unused1] tokens.

SHAP output:

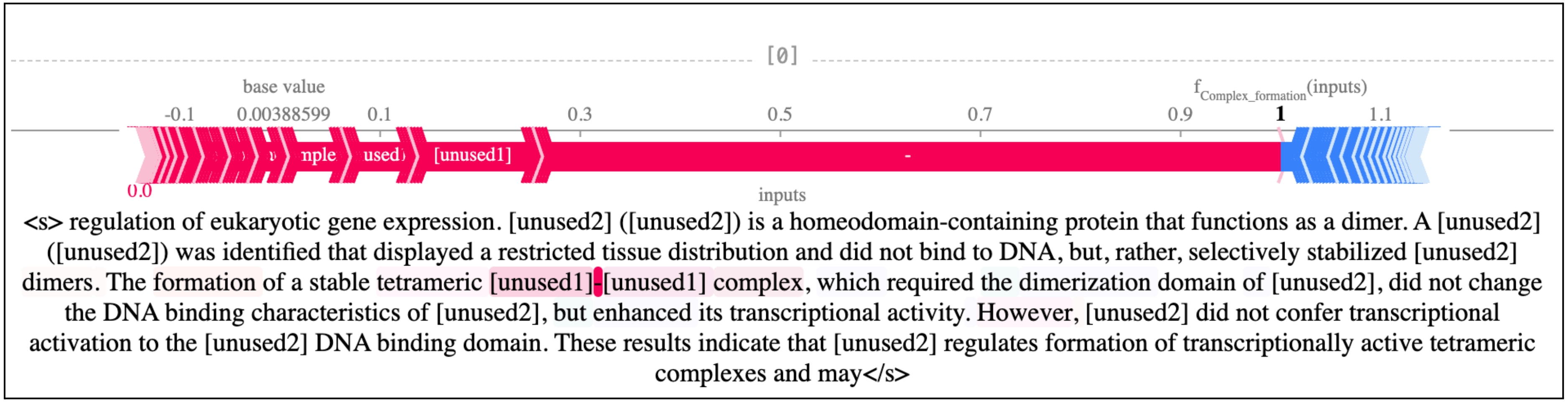

As SHAP result shows, the highest scores belong to '-', [unused1] and 'complex' tokens. This is logical, as removing [unused1] tokens from the input should change the prediction outcome, i.e., the positive label (Complex_formation). This is the reason why we remove [unused1] tokens from the output of SHAP and LIG methods before finding the token with the highest score.

The following is a part of the heatmap, obtained by running the LIG algorithm on the same sentence. As we notice, there are a lot of high hits around the [unused1] tokens, as well as the '-' token and 'Complex' token. As we notice, lower layers in the network (closer to the input), have higher numbers for the '-' and 'complex' tokens, but higher layers (closer to the output), have higher scores for the [unused1] tokens. Also note that the numbers for each row do **not** sum up to 100.

	ĠThe	Ġformation	Ġof	Ġa	Ġstable	Ġtetrameric	Ġ	[unused1]	-	[unused1]	Ġcomplex	,	Ġwhich	Ġrequired	Ġthe	Ġdimerization	Ġdomain
23	0.000	0.000	0.000	0.000	0.000	0.000	0.000	0.000	0.000	0.000	0.000	0.000	0.000	0.000	0.000	0.000	0.000
22	1.531	3.358	4.568	6.362	2.490	5.186	9.769	9.920	9.712	10.513	7.224	3.558	0.717	0.392	0.453	0.432	0.357
21	0.166	2.214	3.321	8.529	1.309	6.305	31.516	34.723	30.925	39.330	12.245	0.316	0.337	0.307	0.248	0.253	0.290
20	-0.600	1.099	1.568	5.060	0.626	1.849	25.754	39.092	50.523	69.007	7.805	-3.590	0.050	0.077	-0.109	-0.139	0.020
19	-1.008	0.586	0.588	1.735	0.354	1.537	19.917	36.754	46.165	77.417	7.517	-7.077	0.158	0.214	0.109	0.164	0.437
18	-0.524	-0.441	-1.075	-1.297	-0.280	-0.317	9.072	58.563	27.092	75.014	-0.713	-3.248	-0.170	0.086	0.310	0.155	0.159
17	-0.422	-0.426	-0.451	-0.341	-0.667	-1.011	2.003	74.995	8.566	65.026	-0.847	-2.921	-0.146	0.017	0.161	-0.123	-0.090
16	-0.732	2.142	1.444	0.787	0.078	-2.099	4.496	73.162	29.060	59.009	2.967	-1.683	0.104	-0.451	-0.003	0.040	-0.058
15	-1.702	1.460	2.146	2.287	2.549	2.247	3.443	59.614	60.360	45.958	21.414	-1.110	-0.469	-0.144	-0.173	0.095	-0.116
14	-2.352	1.425	1.776	2.583	4.649	7.468	7.370	55.175	45.217	58.128	32.410	-1.677	-1.243	-1.132	-0.490	0.127	-0.339
13	-3.934	-0.383	0.963	3.722	4.673	7.945	10.543	35.450	83.759	25.693	26.498	-2.475	-1.211	0.328	-0.527	0.006	-0.497
12	-4.516	0.183	1.168	4.041	7.488	11.607	17.723	24.354	63.085	-1.438	52.631	-3.128	-1.698	-2.574	-1.188	0.504	-0.522
11	-1.618	0.186	-0.054	1.196	2.787	8.131	12.668	41.058	76.323	32.737	16.954	-0.376	-3.265	0.575	0.160	0.121	0.091
10	4.031	2.401	3.598	6.297	7.255	10.582	5.686	-29.344	43.293	-6.746	27.116	-0.452	0.066	0.433	0.205	-0.453	-0.688
9	1.515	2.374	3.740	4.440	8.103	12.772	12.448	14.424	45.284	16.353	58.690	5.942	2.550	-0.851	0.088	-2.133	-1.190
8	0.693	2.635	1.586	1.492	7.467	3.035	15.279	-23.029	65.835	-40.806	42.841	10.222	3.888	3.987	0.170	-2.146	-2.589
7	0.778	2.096	1.154	2.103	6.557	3.309	9.712	-16.883	51.660	-25.789	46.193	3.134	2.661	6.421	0.591	-1.317	-4.095
6	0.866	-1.625	1.156	0.517	6.418	2.126	6.597	-33.233	38.380	28.862	29.817	0.475	-0.008	1.491	-0.498	-2.102	-0.315
5	3.104	1.721	2.600	0.827	3.370	-7.618	11.408	-33.038	49.387	-1.760	56.628	7.129	-2.601	2.540	0.537	-3.642	-3.424
4	1.023	-4.176	-2.734	-0.457	10.280	-10.089	-7.889	-37.395	44.649	2.696	65.498	15.834	1.335	7.550	0.307	-3.733	-0.545
3	-3.857	-0.795	-1.407	1.506	7.086	-1.291	-8.114	-50.828	65.311	-18.844	39.592	-3.819	2.623	-2.522	-0.387	-4.126	-0.715
2	-5.860	-1.289	-4.252	0.215	1.370	2.451	-0.682	-36.576	32.338	-20.239	10.954	-1.307	0.409	1.522	-4.939	-0.090	-1.412
1	-5.761	5.488	-4.735	12.372	7.468	15.919	-35.058	-32.376	47.344	-8.836	26.688	-12.632	-33.351	23.934	5.317	-3.435	-3.390
0	1.458	-2.533	-2.940	-1.930	6.293	9.087	-10.644	14.931	60.572	10.519	41.828	-0.500	0.248	-0.014	2.446	-0.614	0.794
embeddings	-2.905	0.707	4.800	5.773	5.332	-1.487	0.904	-0.386	7.242	6.091	11.238	16.226	-1.650	1.032	-2.435	0.429	0.244

E. Closed class words in trigger word detection

```
CCWords = {'&', "'cause", "'n", "'n'", "'til", 'I', 'a', 'aboard', 'about',
'above', 'across', 'after', 'against', 'ago','albeit', 'all', 'along',
'alongside', 'although', 'always', 'am', 'amid', 'among', 'amongst', 'an',
'and', 'any', 'anybody', 'anyhow', 'anyone', 'anything', 'anytime',
'anyway', 'anywhere', 'are', 'around', 'as', 'astride', 'at','atop', 'be',
'because', 'been', 'before', 'behind', 'being', 'below', 'beneath',
'beside', 'besides', 'between','beyond', 'billion', 'billionth', 'both',
'but', 'by', 'can', 'cannot', 'could', 'de', 'despite', 'did', 'do','does',
'doing', 'done', 'down', 'during', 'each', 'eight', 'eighteen',
'eighteenth', 'eighth', 'eightieth', 'eighty','either', 'eleven',
'eleventh', 'en', 'enough', 'et', 'every', 'everybody', 'everyone',
'everything', 'everywhere','except', 'few', 'fewer', 'fifteen',
'fifteenth', 'fifth', 'fiftieth', 'fifty', 'first', 'five', 'for',
'fortieth','forty', 'four', 'fourteen', 'fourteenth', 'fourth', 'from',
'had', 'has', 'have', 'having', 'he', 'her', 'here', 'hers', 'herself',
'him', 'himself', 'his', 'how', 'hundred', 'hundredth', 'if', 'in',
'inside', 'into', 'is','it', 'its', 'itself', 'least', 'less', 'lest',
'like', 'little', 'many', 'may', 'me', 'might', 'million', 'millionth',
'mine', 'minus', 'more', 'most', 'much', 'must', 'my', 'myself', 'near',
'neither', 'never', 'next', 'nine', 'nineteen', 'nineteenth', 'ninetieth',
'ninety', 'ninth', 'no', 'nobody', 'none', 'nor', 'not', 'nothing',
'notwithstanding', 'now', 'nowhere', 'of', 'off', 'on', 'one', 'oneself',
'onto', 'opposite', 'or', 'our', 'ours', 'ourselves', 'out', 'outside',
'over', 'par', 'past', 'per', 'plus', 'post', 'second', 'seven',
'seventeen', 'seventeenth', 'seventh', 'seventieth', 'seventy', 'shall',
'she', 'should', 'since', 'six', 'sixteen', 'sixteenth', 'sixth',
'sixtieth', 'sixty', 'so', 'some', 'somebody', 'somehow', 'someone',
'something', 'sometime', 'somewhere', 'ten', 'tenth', 'than', 'that',
'the', 'their', 'theirs', 'them', 'themselves', 'then', 'there', 'these',
'they', 'third', 'thirteen', 'thirteenth', 'thirtieth', 'thirty', 'this',
'those', 'though', 'thousand', 'thousandth', 'three', 'through',
'throughout', 'till', 'times', 'to', 'too', 'toward', 'towards', 'twelfth',
'twelve', 'twentieth', 'twenty', 'two', 'under', 'underneath', 'unless',
'unlike', 'until', 'unto', 'up', 'upon', 'us', 'v.', 'versus', 'via',
'vs.', 'was', 'we', 'were', 'what', 'when', 'where', 'whereas', 'whether',
'which', 'while', 'who', 'whom', 'whose', 'why', 'will', 'willing', 'with',
'within', 'without', 'worth', 'would', 'yes', 'yet', 'you', 'your',
'yours', 'yourself', 'yourselves', 'zero'}
```

6. Distribution of entities and relations in the ComplexTome corpus

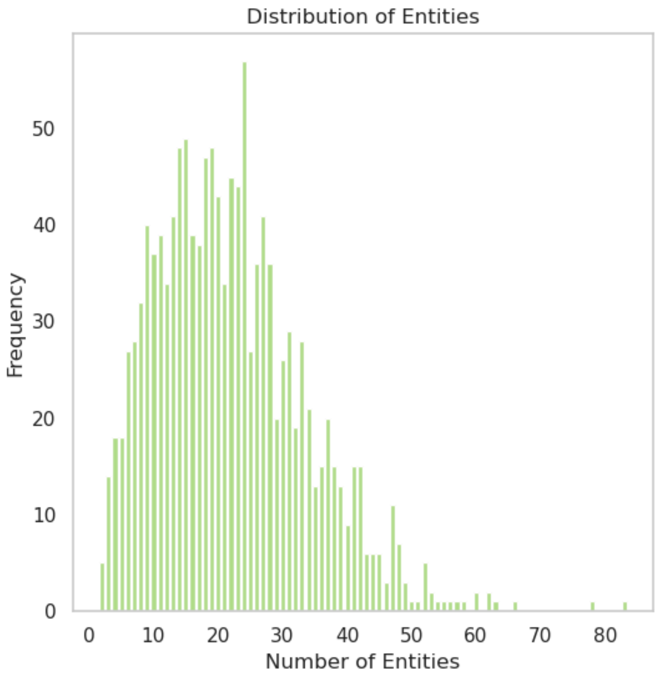

The distribution of number of entities in the ComplexTome corpus. Most of the documents have between 5 and 40 entities in total, corresponding to all NE types we have annotated in ComplexTome (i.e. `Protein`, `Complex`, `Family` and `Chemical`).

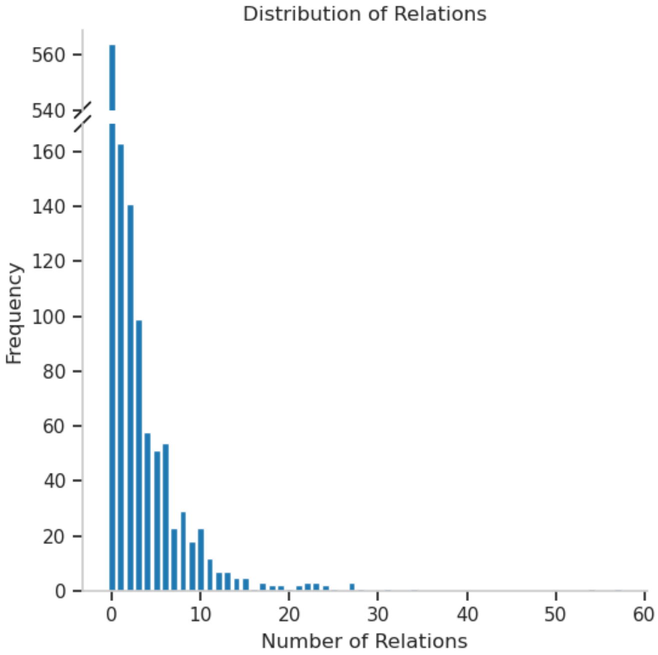

The distribution of the number of relations in the ComplexTome corpus. This distribution is heavily skewed to the left with the vast majority of the documents in ComplexTome having no relation annotations, while for those that have annotations only a few have more than 7 relations in the same document.

7. Table with results of relation extraction error analysis results for the best model on the test set

Screenshots of the errors from BRAT are available via the associated Zenodo project. You can find a link to the Zenodo project in the paper. If you wish to setup a BRAT server to view the errors yourself, please follow the instructions provided through the annotation documentation https://katnastou.github.io/annodoc-physical-protein-interaction-corpus/.
FN: False Negative, FP: False Positive

PubMed ID	Relationship ID	Entity 1	Entity 2	FP/FN	Error Type
8407894	R5	T6	T10	FP	ambiguous keyword
8407894	R7	T7	T10	FP	ambiguous keyword
8407894	R9	T8	T10	FP	ambiguous keyword
8407894	R11	T9	T10	FP	ambiguous keyword
8505295	R7	T9	T10	FP	ambiguous keyword
8505295	R8	T9	T11	FP	ambiguous keyword
8505295	R9	T9	T26	FP	ambiguous keyword
8505295	R10	T9	T27	FP	ambiguous keyword
9195882	R3	T3	T26	FP	ambiguous keyword
9367446	R1	T19	T6	FP	ambiguous keyword
9367446	R2	T20	T6	FP	ambiguous keyword
16908542	R12	T18	T19	FP	ambiguous keyword
19276361	R8	T21	T22	FP	ambiguous keyword
29283431	R4	T8	T9	FP	ambiguous keyword
20797779_6	R1	T8	T5	FP	ambiguous keyword
22958824_7	R2	T8	T9	FP	ambiguous keyword
26651479_12	R3	T1	T2	FP	ambiguous keyword
28515143_17	R3	T19	T20	FP	ambiguous keyword
8407894	R2	T6	T7	FN	ambiguous keyword
8407894	R3	T7	T8	FN	ambiguous keyword
8407894	R4	T8	T9	FN	ambiguous keyword
8407894	R5	T6	T8	FN	ambiguous keyword
8407894	R6	T6	T9	FN	ambiguous keyword
8407894	R7	T7	T9	FN	ambiguous keyword
8407894	R13	T18	T19	FN	ambiguous keyword
9115214	R3	T4	T3	FN	ambiguous keyword
9115214	R4	T1	T2	FN	ambiguous keyword
9195882	R5	T27	T6	FN	ambiguous keyword
9506992	R2	T10	T11	FN	ambiguous keyword

PubMed ID	Relationship ID	Entity 1	Entity 2	FP/FN	Error Type
10433269	R13	T5	T4	FN	ambiguous keyword
21236256	R4	T3	T4	FN	ambiguous keyword
21554248	R19	T21	T22	FN	ambiguous keyword
21554248	R23	T20	T22	FN	ambiguous keyword
22355353	R2	T3	T11	FN	ambiguous keyword
25602519	R7	T18	T19	FN	ambiguous keyword
27751725	R5	T32	T10	FN	ambiguous keyword
27751725	R6	T31	T10	FN	ambiguous keyword
27751725	R7	T30	T10	FN	ambiguous keyword
27751725	R8	T29	T10	FN	ambiguous keyword
29666278	R19	T10	T28	FN	ambiguous keyword
22086907_3	R1	T5	T4	FN	ambiguous keyword
23499533_3	R1	T3	T6	FN	ambiguous keyword
28515143_17	R3	T12	T13	FN	ambiguous keyword
9348293_33	R20	T33	T34	FN	ambiguous keyword
11416152	R14	T51	T22	FP	annotation error
11416152	R15	T24	T53	FP	annotation error
12151385	R9	T13	T18	FP	annotation error
16908542	R11	T13	T29	FP	annotation error
17194709	R7	T7	T25	FP	annotation error
17194709	R8	T24	T25	FP	annotation error
20463880	R1	T10	T13	FP	annotation error
20463880	R2	T11	T13	FP	annotation error
20463880	R3	T12	T13	FP	annotation error
24498436	R14	T27	T11	FP	annotation error
25938661_25	R1	T6	T7	FP	annotation error
11874917	R3	T12	T14	FN	annotation error
11897782	R6	T5	T6	FN	annotation error
11897782	R11	T10	T12	FN	annotation error
11897782	R12	T11	T12	FN	annotation error
16738327	R3	T7	T8	FN	annotation error
16912044	R5	T23	T4	FN	annotation error
16912044	R9	T26	T9	FN	annotation error
16912044	R10	T9	T10	FN	annotation error
14562105	R5	T30	T17	FP	co-reference resolution
16177062	R13	T18	T21	FP	co-reference resolution

PubMed ID	Relationship ID	Entity 1	Entity 2	FP/FN	Error Type
16177062	R14	T18	T42	FP	co-reference resolution
16209941	R24	T32	T5	FP	co-reference resolution
16209941	R26	T32	T6	FP	co-reference resolution
16209941	R28	T32	T7	FP	co-reference resolution
16209941	R30	T32	T8	FP	co-reference resolution
16260776	R1	T5	T6	FP	co-reference resolution
16260776	R2	T5	T7	FP	co-reference resolution
20406818	R8	T25	T27	FP	co-reference resolution
23022657_23	R2	T6	T8	FP	co-reference resolution
10446169	R12	T12	T13	FN	co-reference resolution
15485920	R6	T19	T20	FN	co-reference resolution
15485920	R9	T24	T25	FN	co-reference resolution
16177062	R16	T21	T42	FN	co-reference resolution
16908542	R1	T5	T6	FN	co-reference resolution
16908542	R3	T1	T2	FN	co-reference resolution
16908542	R4	T1	T3	FN	co-reference resolution
20406818	R6	T26	T27	FN	co-reference resolution
23022657_23	R1	T9	T8	FN	co-reference resolution
1719979	R1	T6	T7	FP	convoluted text excerpt
1719979	R2	T6	T8	FP	convoluted text excerpt
9858532	R8	T11	T12	FP	convoluted text excerpt
15449939	R6	T18	T19	FP	convoluted text excerpt
17435760	R1	T2	T3	FP	convoluted text excerpt
18317453	R3	T3	T4	FP	convoluted text excerpt
24498436	R15	T13	T29	FP	convoluted text excerpt
25569479_19	R1	T12	T13	FP	convoluted text excerpt
9195882	R4	T27	T28	FP	convoluted text excerpt
10585430	R6	T31	T26	FP	convoluted text excerpt
11416152	R10	T10	T38	FP	convoluted text excerpt
11416152	R11	T41	T15	FP	convoluted text excerpt
11416152	R12	T42	T16	FP	convoluted text excerpt
11416152	R13	T20	T50	FP	convoluted text excerpt
12135708	R7	T18	T19	FP	convoluted text excerpt
11520069	R2	T13	T3	FN	convoluted text excerpt
11520069	R4	T14	T3	FN	convoluted text excerpt
11520069	R5	T15	T3	FN	convoluted text excerpt

PubMed ID	Relationship ID	Entity 1	Entity 2	FP/FN	Error Type
11520069	R6	T14	T4	FN	convoluted text excerpt
11520069	R7	T15	T4	FN	convoluted text excerpt
11520069	R8	T13	T4	FN	convoluted text excerpt
11867544	R2	T19	T5	FN	convoluted text excerpt
12151385	R14	T21	T34	FN	convoluted text excerpt
12151385	R15	T21	T22	FN	convoluted text excerpt
14670962	R4	T21	T9	FN	convoluted text excerpt
15449939	R11	T16	T15	FN	convoluted text excerpt
16209941	R17	T42	T18	FN	convoluted text excerpt
16209941	R18	T42	T19	FN	convoluted text excerpt
19228687	R1	T1	T2	FN	convoluted text excerpt
19228687	R2	T4	T3	FN	convoluted text excerpt
21236256	R15	T11	T12	FN	convoluted text excerpt
21807881	R2	T3	T2	FN	convoluted text excerpt
21827752	R5	T12	T5	FN	convoluted text excerpt
9348293_33	R11	T17	T18	FN	convoluted text excerpt
9348293_33	R12	T16	T18	FN	convoluted text excerpt
9348293_33	R17	T27	T28	FN	convoluted text excerpt
7524088	R2	T4	T6	FN	convoluted text excerpt
8505295	R9	T14	T16	FN	convoluted text excerpt
8505295	R10	T17	T16	FN	convoluted text excerpt
10770935	R2	T3	T7	FN	convoluted text excerpt
21347367	R5	T6	T15	FN	convoluted text excerpt
8521815	R4	T8	T27	FN	convoluted text excerpt
10446169	R11	T21	T11	FN	convoluted text excerpt
11416152	R10	T13	T40	FN	convoluted text excerpt
11416152	R14	T43	T16	FN	convoluted text excerpt
11416152	R15	T43	T44	FN	convoluted text excerpt
11521196	R1	T15	T14	FN	convoluted text excerpt
11521196	R2	T15	T12	FN	convoluted text excerpt
12151385	R8	T17	T15	FN	convoluted text excerpt
17572495	R10	T22	T20	FN	convoluted text excerpt
21554248	R9	T11	T9	FN	convoluted text excerpt
21554248	R10	T11	T35	FN	convoluted text excerpt
23533635	R4	T28	T6	FN	convoluted text excerpt
23533635	R8	T28	T8	FN	convoluted text excerpt

PubMed ID	Relationship ID	Entity 1	Entity 2	FP/FN	Error Type
8505295	R11	T22	T23	FP	rare keyword
14562105	R4	T26	T8	FP	rare keyword
15223	R1	T1	T2	FN	rare keyword
2002555	R2	T5	T4	FN	rare keyword
10666337	R6	T13	T6	FN	rare keyword
11520069	R13	T18	T8	FN	rare keyword
11520069	R14	T18	T7	FN	rare keyword
11713274	R5	T27	T28	FN	rare keyword
11897782	R3	T1	T3	FN	rare keyword
16209941	R22	T44	T23	FN	rare keyword
16717095	R13	T27	T9	FN	rare keyword
16717095	R14	T27	T8	FN	rare keyword
16908542	R13	T14	T30	FN	rare keyword
16908542	R14	T15	T30	FN	rare keyword
16912044	R11	T9	T11	FN	rare keyword
17194709	R13	T21	T5	FN	rare keyword
17194709	R14	T21	T22	FN	rare keyword
18628300	R3	T9	T12	FN	rare keyword
19276361	R5	T2	T32	FN	rare keyword
20129058	R8	T1	T2	FN	rare keyword
20129058	R10	T12	T13	FN	rare keyword
20129058	R11	T14	T15	FN	rare keyword
23533635	R11	T26	T11	FN	rare keyword
25602519	R3	T15	T16	FN	rare keyword
27120157	R29	T22	T23	FN	rare keyword

8. Table with results of trigger word detection error analysis results for the best model on the test set

Screenshots of the errors from BRAT are available via the associated Zenodo project. You can find a link to the Zenodo project in the paper. If you wish to setup a BRAT server to view the errors yourself, please follow the instructions provided through the annotation documentation https://katnastou.github.io/annodoc-physical-protein-interaction-corpus/.
The first part of the results is the PMID of the document (e.g. 10080948) followed by the two entities (e.g. T13 & T14 for the 2nd cell in the first column) for which the trigger words annotations have been made in the provided text snippet. *4 examples (Table B) are counted as TP due to acceptable alternative annotations detected by the annotator and thus only 17 are counted as FP+FN
FN: False Negative, FP: False Positive

A) Correct trigger word detected in documents with multiple triggers (TP) (**Total count: 63)**

PMID_EID1_EID2	detected	not detected
10080948_T13_T14	-	interaction
10096561_T28_T29	-	interactions
10229231_T11_T12	/	interaction
10229231_T3_T4	/	interaction
10229231_T3_T5	/	interaction
10395652_T18_T8	complex	recruited
10395652_T19_T8	complex	recruited
10395652_T5_T6	associates	complex
10704443_5_T2_T3	complex	comprises
10704443_5_T2_T4	complex	comprises
10704443_5_T2_T5	complex	comprises
10704443_5_T3_T4	complex	comprises
10704443_5_T3_T5	complex	comprises
10704443_5_T4_T5	complex	comprises
12024017_T13_T2	/	complex
12024017_T21_T10	/	complex
12897057_T11_T12	complex	/
12897057_T14_T15	/	complex
12897057_T18_T19	/	complex
15225555_T12_T13	targets	yeast two-hybrid

15225555_T12_T14	targets	yeast two-hybrid
1700011_T5_T6	/	heterodimers
1700011_T7_T8	/	heterodimers
1763325_T6_T7	-	complex
19141288_18_T1_T2	complex	/
19141288_18_T7_T8	complex	/
19141288_18_T20_T21	saturation	Kd
19656901_T38_T8	-	associations
19656901_T7_T37	-	associations
20541251_2_T7_T12	interaction	-
21685921_32_T23_T1	-	complexes
21685921_32_T24_T3	-	complexes
22210188_T14_T5	partner	yeast two-hybrid
22210188_T14_T6	partner	yeast two-hybrid
22210188_T4_T5	partner	yeast two-hybrid
22210188_T4_T6	partner	yeast two-hybrid
22210188_T7_T8	interaction	immunoprecipitation
22675546_10_T11_T2	coimmunoprecipitated	complex
22675546_10_T14_T4	complex	-
22675546_10_T1_T2	coimmunoprecipitated	complex
22675546_10_T9_T2	coimmunoprecipitated	complex
24465968_22_T8_T9	-	complex
24498054_T19_T1	/	complex
24498054_T22_T6	/	complex
26996158_T15_T16	-	complex
27044741_T33_T25	-	complex
7492771_T11_T12	/	heterodimers
7492771_T15_T16	/	heterodimers
7492771_T5_T6	/	heterodimers
7537762_T10_T11	binding	ligand
8098618_T18_T19	-	heterodimer
8098618_T21_T22	-	heterodimer
8626752_T1_T2	association	complexes
8626752_T1_T3	association	complexes

8626752_T6_T7	-	heterocomplexes
9512491_T17_T18	-	complex
9512491_T17_T19	-	-<br>complex
9512491_T18_T19	-	complex
9512491_T7_T17	-	complex
9512491_T7_T18	-	-<br>complex
9512491_T7_T19	-	-<br>complex
9733846_T11_T12	-	interaction
9733846_T15_T16	-	interaction

B) Word detected not a trigger (FP+FN) (**Total count: 21)**

PMID_EID1_EID2	detected	not detected
12093729_T4_T41	MP	in<br>complex
12093729_T6_T41	MP	in<br>complex
1700011_T3_T4	together	heterodimeric
17452446_T4_T16	forms	complex
17452446_T4_T17	forms	complex
17452446_T4_T5	forms	complex
17452446_T4_T6	forms	complex
17452446_T8_T9	components	complex
19150429_T1_T2	-	interacting
19217404_T18_T19	form	hexameric
19217404_T18_T21	form	hexameric
19217404_T19_T21	form	hexameric
20541251_2_T10_T6*	target	bind<br>complex
20541251_2_T17_T1	binding	recruitment
20541251_2_T8_T6*	target	bind<br>complex
22904036_T10_T13	including	interacted
22904036_T11_T13	including	interacted
22904036_T3_T4	including	interacting partners
22904036_T3_T5	including	interacting partners

8626752_T12_T13*	-	complex
9751051_T1_T2*	complex	bound

C) No trigger word detected in documents with one trigger word (FN) (**Total count: 34)**

PMID_EID1_EID2	not detected
15314162_T9_T10	binding
17452446_T11_T1	interaction
17452446_T11_T12	interaction
17452446_T12_T1	interaction
19150429_T6_T8	interacts
19217404_T22_T26	receive
19414608_38_T7_T8	interaction
20541251_2_T11_T15	recruitment
21063388_2_T7_T9	interacting
21063388_2_T9_T10	interacting
21907821_T10_T38	binding
21907821_T10_T39	binding
22056990_T10_T3	target
22160715_T8_T4	binding
22160715_T8_T5	binding
22160715_T8_T6	binding
22579246_3_T1_T13	interact
22579246_3_T1_T3	interact
22579246_3_T2_T13	interact
22579246_3_T2_T3	interact
26675234_T12_T14	binds
27044741_T23_T24	-
27044741_T28_T8	-
27044741_T30_T14	-
27044741_T5_T6	-
27044741_T7_T4	-
8649779_T10_T11	subunits
8649779_T10_T12	subunits
8649779_T10_T13	physically interact
8649779_T11_T12	subunits
9171108_T3_T4	associated

9356494_T9_T11	associated
9512491_T27_T28	effector
9990065_T10_T11	radioligand binding

D) No trigger word detected in documents with multiple trigger words (FN) **Total count: 4**

PMID_EID1_EID2	not detected
14707132_T28_T30	two-hybrid screen<br>target
14707132_T29_T30	two-hybrid screen<br>target
22675546_10_T4_T5	recruitment<br>complex
27462423_T11_T12	/<br>complex

Correct trigger word detected in documents with one trigger word (TP) (**Total count: 152)**

10080948_T10_T11, 10080948_T1_T2, 10080948_T3_T4, 10080948_T6_T7, 10080948_T8_T9, 10395652_T9_T20, 11959841_T18_T4, 12093729_T10_T11, 12093729_T10_T44, 12093729_T17_T45, 12223469_T14_T15, 12223469_T16_T17, 12223469_T16_T18, 12223469_T1_T2, 12223469_T24_T25, 12223469_T30_T31, 12223469_T6_T7, 12223469_T9_T10, 14973287_T17_T20, 14973287_T17_T5, 14973287_T17_T6, 15107829_T3_T4, 15107829_T3_T5, 15107829_T8_T9, 15150273_T23_T24, 15150273_T29_T2, 15150273_T37_T11, 15150273_T41_T16, 15314162_T8_T9, 16546083_T19_T20, 16546083_T5_T6, 16787403_T19_T5, 16787403_T6_T7, 17452446_T14_T4, 17452446_T15_T4, 17452446_T5_T6, 17666399_T13_T30, 17666399_T7_T22, 17666399_T7_T23, 18029035_T14_T3, 18029035_T4_T6, 18029035_T4_T7, 18455122_T5_T6, 18455122_T5_T7, 19141288_18_T14_T15, 19141288_18_T3_T4, 19150429_T11_T2, 19150429_T11_T3, 19150429_T1_T3, 19150429_T6_T7, 19217404_T11_T12, 19217404_T11_T14, 19217404_T22_T23, 19217404_T26_T28, 19414608_38_T7_T9, 19414608_38_T8_T9, 20472562_T12_T15, 20472562_T12_T16, 20472562_T13_T15, 20472562_T13_T16, 20472562_T18_T20, 20472562_T19_T20, 20541251_2_T11_T22, 20541251_2_T17_T18, 20541251_2_T24_T9, 21063388_2_T12_T13, 21063388_2_T15_T16, 21063388_2_T19_T20, 21063388_2_T3_T5, 21063388_2_T3_T6, 21063388_2_T7_T8, 21985068_5_T2_T17, 21985068_5_T2_T3, 21985068_5_T2_T4, 21985068_5_T2_T5, 22009753_T9_T10, 22160715_T16_T1, 22210188_T11_T12, 22210188_T1_T13, 22210188_T1_T3, 22210188_T2_T13, 22210188_T2_T3, 22210188_T9_T10, 22675546_10_T17_T7, 22675546_10_T18_T7, 22675546_10_T3_T12, 22675546_10_T3_T13, 22675546_10_T6_T15, 22675546_10_T8_T20, 22842725_T3_T16, 22842725_T3_T4, 22904036_T10_T12, 22904036_T10_T14, 22904036_T11_T12, 22904036_T11_T14, 22904036_T9_T10, 22904036_T9_T11, 23446637_T1_T2, 23446637_T8_T16, 23446637_T8_T9, 23498974_T17_T18, 23498974_T17_T19, 24498054_T20_T2, 26675234_T12_T13, 26972597_T27_T28, 26972597_T32_T35, 26972597_T32_T36, 26996158_T10_T11, 26996158_T14_T15, 27334688_T6_T7, 27462423_T10_T11, 27462423_T10_T12, 27462423_T9_T18, 7492771_T1_T2, 7747417_T7_T8, 7747417_T9_T10, 8108127_T20_T21, 8108127_T20_T22, 8156587_T10_T5, 8626752_T10_T11, 8626752_T14_T15, 8626752_T14_T16, 8626752_T17_T19, 8626752_T18_T19, 8626752_T8_T9, 8649779_T11_T13, 8649779_T12_T13, 9171108_T11_T12, 9171108_T13_T14, 9171108_T1_T4, 9171108_T2_T4, 9171108_T9_T10, 9233773_T14_T12, 9233773_T16_T5, 9233773_T16_T6, 9353251_T12_T13, 9353251_T8_T9, 9356494_T12_T13, 9356494_T1_T2, 9356494_T9_T10, 9512491_T25_T26, 9751051_T3_T4, 9794375_T16_T17, 9794375_T16_T18, 17452446_T16_T5, 17452446_T16_T6, 17452446_T17_T5, 17452446_T17_T6, 19141288_18_T18_T19, 26996158_T14_T16, 9233773_T14_T13, 9233773_T14_T4

9. Text-mining results for the physical interaction between two proteins in STRING v11.5 and v12

Example of text excerpts supporting a text-mined physical protein interaction between TrpA and TrpB from the web interfaces of STRING v11.5 to v12. The same publication is shown for better comparison between the two versions. Trigger words are highlighted in v12.

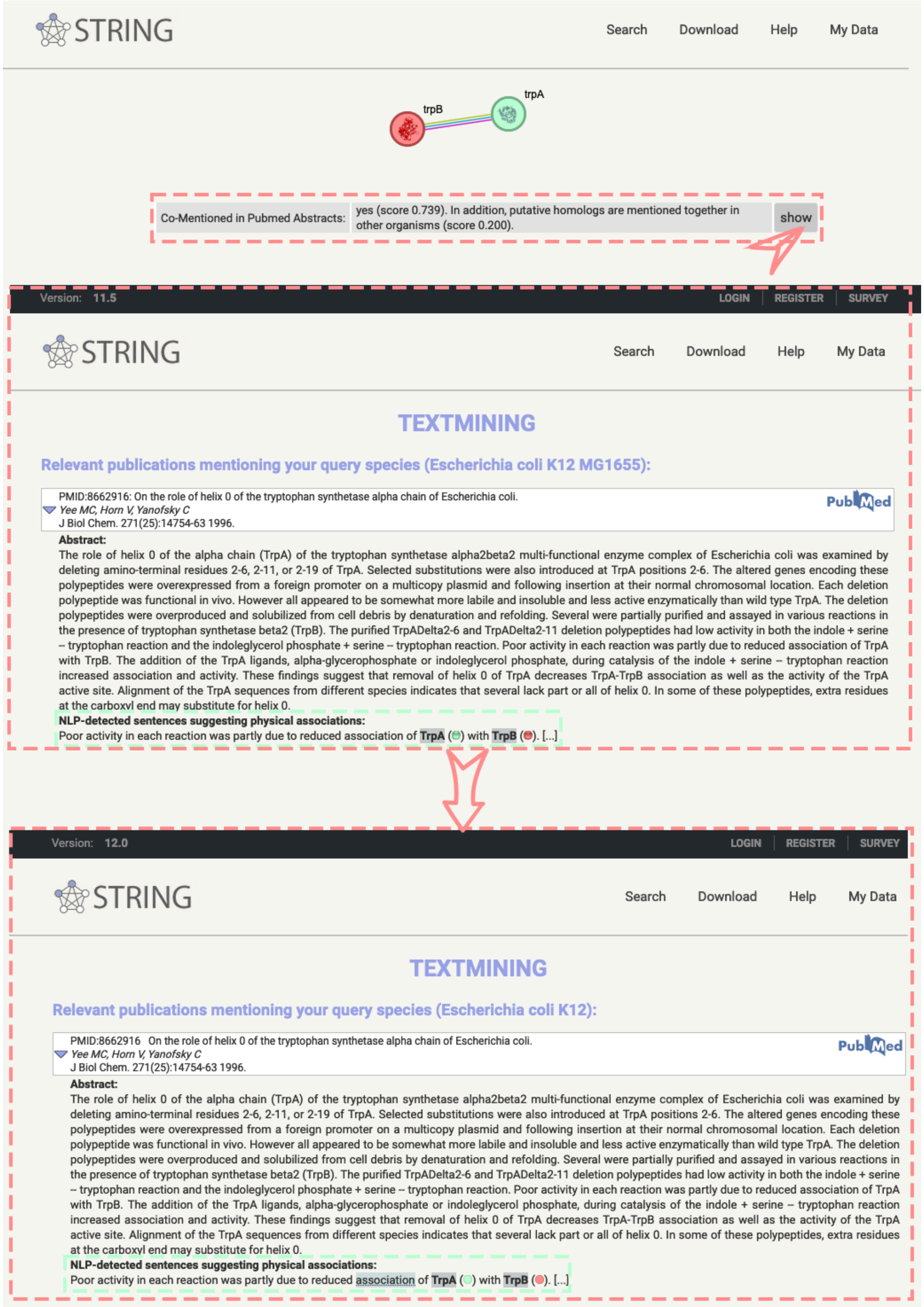

References:

[1] A. Vaswani *et al.*, ‘Attention is All you Need’, in *Advances in Neural Information Processing Systems*, 2017, vol. 30.

[2] P. Lewis, M. Ott, J. Du, and V. Stoyanov, ‘Pretrained Language Models for Biomedical and Clinical Tasks: Understanding and Extending the State-of-the-Art’, in *Proceedings of the 3rd Clinical Natural Language Processing Workshop*, 2020, pp. 146–157.

[3] A. Miranda-Escalada *et al.*, ‘Overview of DrugProt task at BioCreative VII: data and methods for large-scale text mining and knowledge graph generation of heterogeneous chemical–protein relations’, *Database*, vol. 2023, p. baad080, 11 2023.

[4] L. Weber, M. Sänger, S. Garda, F. Barth, C. Alt, and U. Leser, ‘Chemical–protein relation extraction with ensembles of carefully tuned pretrained language models’, *Database*, vol. 2022, p. baac098, 11 2022.

[5] J. Luoma *et al.*, ‘S1000: a better taxonomic name corpus for biomedical information extraction’, *Bioinformatics*, vol. 39, no. 6, p. btad369, 06 2023.

[6] J. Lee *et al.*, ‘BioBERT: a pre-trained biomedical language representation model for biomedical text mining’, *Bioinformatics*, vol. 36, no. 4, pp. 1234–1240, 09 2019.

[7] H.-C. Shin *et al.*, ‘BioMegatron: Larger Biomedical Domain Language Model’, in *Proceedings of the 2020 Conference on Empirical Methods in Natural Language Processing (EMNLP)*, 2020, pp. 4700–4706.

[8] Y. Liu *et al.*, ‘RoBERTa: A Robustly Optimized BERT Pretraining Approach’. 2020.

[9] J. Devlin, M.-W. Chang, K. Lee, and K. Toutanova, ‘BERT: Pre-training of Deep Bidirectional Transformers for Language Understanding’, in *Proceedings of the 2019 Conference of the North American Chapter of the Association for Computational Linguistics: Human Language Technologies, Volume 1 (Long and Short Papers)*, 2019, pp. 4171–4186.
